# Institutional CT dose reduction: A retrospective evaluation of dose indices from 2015–2024

**DOI:** 10.1002/acm2.70823

**Published:** 2026-10-05

**Authors:** Jayse M Weaver, Afrouz Ataei, Nicole E Murphy, Daniel R L'Heureux, Mark P Supanich

**Affiliations:** ^1^ Division for Medical Physics Department of Radiology and Nuclear Medicine Rush University Medical Center Rush Medical College Chicago Illinois USA; ^2^ Division of Body Imaging Department of Diagnostic Radiology and Nuclear Medicine Rush University Medical Center Rush Medical College Chicago Illinois USA

**Keywords:** computed tomography, dose indices, dose reduction

## Abstract

**Background:**

Growing attention to radiation risks and dose reduction in CT imaging creates opportunities to present and discuss institutional dose trends and contributing factors.

**Purpose:**

To provide an evaluation of 10‐year trends in CT dose indices (CTDI_vol_ and DLP) from a multi‐site and multi‐scanner institution, with the hypothesis that doses have continued to decrease since the last national assessment of medical imaging doses due to scientific and technological improvements.

**Methods:**

Data from a radiation dose index management system (Radimetrics, Bayer) were used to analyze diagnostic CT examinations from January 2015 to December 2024, stratified by procedure. Dose indices for the ten most utilized procedures were evaluated over the 10‐year period using multivariable linear regression, adjusted for patient weight and age, to estimate annual percent change in dose. Procedures were further stratified by scanner model with quarterly median dose indices for qualitative investigation of trends. To investigate the relationship between dose reductions and image quality, archived annual ACR phantom images were retrieved and evaluated using a semi‐automated software (https://ctpro.net/home).

**Results:**

Over the 10‐year period, 695,575 diagnostic CT examinations of 235,233 patients were performed on 18 unique scanners (nine models) from three manufacturers. Multivariable linear regression, adjusted for patient weight and age, showed significant decreases in dose indices over time for most high‐usage procedures. Model‐specific qualitative analyses suggest that acquisition of new scanners, technique changes, and reductions in multiphase imaging were temporally associated with the observed decreases in dose indices. Additionally, the detectability index of low‐contrast ACR phantom objects increased on new scanner models despite lower CTDI_vol_.

**Conclusions:**

A retrospective review of CT exams demonstrated decreasing CTDI_vol_ and DLP across multiple frequently performed procedures, coinciding with known technique and technological changes.

## 1 INTRODUCTION

At the beginning of the 21^st^ century, medical imaging using computed tomography (CT) and magnetic resonance imaging was considered by physicians to be the most important recent medical innovation.^1^ CT is the reference standard for many applications, such as the visualization of kidney stones,^2^ rapid evaluation of intracranial hemorrhage,^3^ and the imaging of lung disease,^4^ despite the presumed risks associated with exposure to low levels of ionizing radiation.^5^ The past several decades has seen an increased interest in reducing and studying the effects of exposure to ionizing radiation in the diagnostic energy range following the publication of a theoretical analysis of cancer risk in pediatric patients after receiving a CT exam.^6^ Technical advancements such as tube current modulation, tube potential optimization, beam shaping and collimation, improved detector technology, and new reconstruction techniques have all contributed to dose reduction in the current era of CT.^7^, ^8^ More recently, the use of novel photon‐counting detectors has been shown to enable dose reduction across applications.^9^ Dose reduction can also be achieved on existing scanners through ongoing technique optimization efforts by medical physicists, radiologic technologists, and radiologists to achieve adequate image quality using the lowest doses possible.^10^

In the United States, assessment of effective dose from medical procedures by the National Council on Radiation Protection and Measurements (NCRP) found a sixfold increase in effective dose between 1980 and 2006 that seemed largely driven by the proliferation of CT examinations.^11^, ^12^ However, a follow‐up assessment of the years between 2006 and 2016 found that despite an increase in number of scans, the estimated average annual individual effective dose for CT decreased.^13^, ^14^ Furthermore, discussion regarding the potential role of CT in increasing cancer risk is ongoing, although a causal relationship is yet to be established.^15^, ^16^, ^17^, ^18^, ^19^, ^20^

Few recent publications have focused on dose and dose reductions at the institutional level. Rizzo et al demonstrated a reduction in CTDI_vol_ and DLP for chest and abdominal exams following an upgrade to newer‐model scanners.^21^ Rehani et al^22^ and Shokoohi et al^23^ both evaluated the incidence of high‐dose exams (defined as an effective dose ≥ 50 mSv) at their shared institution, finding increases in the percentage of high‐dose diagnostic and interventional exams over a 10‐year period^22^ and in the emergency department over a 4‐year period.^23^

The purpose of this work is to provide insight into our institution‐level dose changes by analyzing CT scanner output metrics (CTDI_vol_ and DLP) for the most utilized diagnostic procedures over a 10‐year period (2015–2024) and identify causes for changes, such as the installation of new CT scanners, major software upgrades, and protocol technique modifications, with the hypothesis that our institutional dose levels have continued to decrease in line with anticipated national trends.

## 2 METHODS

### 2.1 Data collection

This retrospective analysis of dose indices was conducted at an academic medical center and received institutional review board approval with waiver of written informed consent. The data were obtained from Radimetrics (Bayer Healthcare), an automated radiation dose index monitoring (RDIM) software application that extracts relevant information from DICOM objects and the Radiation Dose Structured Report (RDSR). All CT exams performed in the 10‐year span of 2015 to 2024 were included in the RDIM report. Exams spanned multiple care settings within a single metropolitan area: an academic medical institution, a community hospital, and multiple outpatient clinics. Here, “exam” is defined as a collection of one or more CT acquisitions in one session with a unique accession number, and “procedure” is defined as the ordered procedure associated with the exam and accession number (e.g. CT Abdomen Without Contrast, CT Brain With Contrast). For each exam, the following data were exported from Radimetrics: accession number, date performed, device model and name, study description, protocol name, procedure name, scan region, acquisition count, age at scan, CTDI_vol_ and DLP for head and body, and patient weight, height, BMI, water equivalent diameter (WED),^24^, ^25^ and geometrical (effective) diameter. Exams without an accession number were removed to exclude non‐patient phantom scans.

While Radimetrics does provide estimates of SSDE and effective dose, CTDI_vol_ and DLP were chosen as the metrics to investigate as patient size estimates and thus SSDE and effective dose (which, in Radimetrics, is estimated by matching patient diameter to phantom sizes) were either missing or inconsistent for portions of the 10‐year period. Effective dose was not considered for analysis for a number of additional reasons. First, effective dose is a biological estimate of risk to a population exposed to ionizing radiation, as such, estimating risk for an individual based on the population‐based organ weighting factors is illogical. Second, effective dose estimated for patient populations using k‐factors is simply a conversion coefficient applied to scanner output. Third, more advanced approaches to estimate effective doses to patient populations using size, age and gender‐matched phantoms and look‐up tables require patient size estimates, which as stated earlier, were missing or inconsistent for portions of the 10‐year period.

### 2.2 Data grouping

The equipment label and procedure name for each exam were used to distinguish diagnostic exams from other CT imaging contexts (primarily interventional and PET‐CT). Procedure names were harmonized using a manual review process based on body region(s) and use of contrast to consolidate names into generic procedure categories. For example, “CT ABDOMEN WO IV CONT” and “CT ABDOMEN WITHOUT IV CONTRAST” were both assigned the generic procedure name “CT ABD WO”. In some instances, procedure name was missing, and the study description was sufficient to map to a generic procedure name. This harmonization was necessary to reduce artificial fragmentation of procedure counts due to naming differences and inconsistencies across sites and over time. Exams were then stratified by generic procedure names, and analysis of dose index trends was performed for the ten most‐utilized diagnostic procedures. The procedure mapping dictionary is provided in Table S‐1.

### 2.3 Statistical analyses

For each of the ten most utilized procedures, the dose indices were modeled as a function of time using multivariable linear regression. As dose indices are right‐skewed, the natural logarithm of each dose index was used as the dependent variable. For each procedure, the following model was fit:

$$\begin{array}{cc} \; & \log\left(Dose\;Index\right)=\beta_{0}+\beta_{1}\left(Time\right)+\beta_{2}\left(Patient\;Weight\right)\\ & +\beta_{3}\left(Patient\;Age\right)+\varepsilon \end{array}$$

where Time was coded as the number of days elapsed since January 1, 2015, and patient weight (kg) and age (years) were included as covariates. Linear models were fit separately for each protocol and for each dose index (CTDI_vol_, DLP), yielding 20 total models. The coefficient for Time (β_1_) was used to calculate the estimated annual percent change in dose metric, and the *p*‐value used to assess statistical significance of the time trend. For trend visualization, monthly median values of each dose index were plotted alongside the model‐derived trend line.

Scanner model was not included as a covariate due to its lack of independence from time, arising from multiple scanners being introduced and decommissioned during the study period. All procedures were further stratified by scanner model, with dose index medians calculated quarterly for each scanner model to better capture scanner‐specific dose changes. Quarterly median values for each model were plotted for qualitative interpretation of model‐specific trends.

Additionally, for each procedure and month, the mean number of acquisitions per exam was calculated. The Pearson correlation was calculated between the monthly mean acquisition count and time (defined here as months since January 1, 2015). A negative Pearson correlation coefficient with a *p*‐value < 0.05 would suggest a decrease in the number of multiphase imaging or exams with repeated series.

Lastly, as an increase in average population size (weight or diameter) may result in an increase in mean or median CTDI_vol_ and DLP due to the relationship between patient size and radiation output. The average patient weight and average patient diameter were calculated for each year from exams with available weight and diameter, and linear regression was used to investigate yearly changes in patient body habitus.

All analyses were conducted using the R language (version 4.5.1, The R Project for Statistical Computing) in RStudio (Posit Software, PBC, Boston, MA).

### 2.4 Annual phantom dose and image quality

To illustrate the effect of new scanner technology on dose reduction, images of the ACR CT accreditation phantom scanned with an adult abdomen protocol were retrieved for same‐manufacturer diagnostic scanners in use in 2015 (SOMATOM Sensation 64, Definition AS, Definition Flash), and the newest scanners in use in 2024 (SOMATOM Drive, NAEOTOM Alpha). As the newly acquired scanners shared a manufacturer, comparisons were made between older scanners from the same manufacturer to avoid inter‐manufacturer comparisons. Additionally, for the older scanners, only data from 2015 was included as the acquisition protocol for the abdominal ACR images did not change from 2015–2024.

Image quality metrics including low contrast detectability, average frequency and noise level of the noise power spectrum, and the maximum visible high‐contrast bar pattern were obtained from the semi‐automated CTPro software (https://ctpro.net/home).^26^ Low contrast detectability was measured by a Channelized Hotelling Observer (CHO) which provides the detectability index (d’), a figure of merit for low contrast detectability that measures the separability of the presence and absence of a signal.^27^, ^28^, ^29^ The default CHO calculation parameters were used: a 4‐channel Gabor filter with two low passbands ([1/64, 1/32] and [1/32, 1/16]) and two orientations (0 and π/2).

## 3 RESULTS

### 3.1 Dataset characteristics

Our dataset includes 695,575 diagnostic CT exams of 235,233 patients between the start of 2015 and end of 2024. All exams were included regardless of age, with pediatric (age at scan < 18 years) exams comprising just 1.35% of the total dataset. Exams were performed on 18 scanners, consisting of 9 unique scanner models. A summary of the scanners, including the time frame used and total exams, is shown in Table 1. Major changes to the scanner fleet occurred in 2023, when a Siemens Sensation 64, GE HealthCare BrightSpeed, and Philips Brilliance 64 were decommissioned and replaced with a Siemens NAEOTOM Alpha and three Siemens SOMATOM Drive scanners.

**TABLE 1:** Summary of CT scanners used across the institution for diagnostic imaging during the time period of January 1st, 2015, to December 31st, 2024.

ID	First Exam	Last Exam	Total Exams
SOMATOM Definition AS #1	1/1/2015	5/14/2019	34119
SOMATOM Definition Flash #1	1/1/2015	12/28/2024	160909
SOMATOM Definition Flash #2	1/1/2015	12/31/2024	80445
Sensation 64 #1	1/1/2015	7/17/2023	18543
SOMATOM Definition AS #2	1/2/2015	12/31/2024	34993
SOMATOM Definition Flash #3	1/2/2015	12/31/2024	64910
BrightSpeed #1	1/3/2015	2/6/2023	56932
Brilliance 64 #1	1/5/2015	2/28/2023	57529
SOMATOM Definition AS #3	1/6/2015	12/31/2024	19803
Revolution EVO #1	10/1/2018	12/31/2024	17152
Revolution EVO #2	1/14/2019	12/31/2024	22669
Revolution CT #1	2/18/2019	12/31/2024	75727
BrightSpeed #2	6/25/2022	12/13/2024	376
NAEOTOM Alpha #1	2/7/2023	12/31/2024	12749
SOMATOM Drive #1	2/7/2023	12/31/2024	12846
SOMATOM Drive #2	2/7/2023	12/31/2024	13019
SOMATOM Drive #3	9/26/2023	12/31/2024	12522
SOMATOM Drive #4	10/28/2024	12/31/2024	332

### 3.2 Procedure prevalence

The top ten most frequently used procedures across all diagnostic exams are listed in Table 2 with the number of each exam performed and proportion of all 695,575 exams. Non‐contrast brain (CT BRAIN WO, 22%), abdomen/pelvis with contrast (CT ABD/PELV W, 11%), and non‐contrast chest (CT CHEST WO, 9%) exams were the three most frequent procedures. The ten most frequently utilized procedures included one angiography procedure (CTA HEAD W, 3%), and no extremity procedures.

**TABLE 2:** Summary of most frequently used diagnostic procedures from 2015–2024 with count and percentage of total exams (out of 695,575).

Diagnostic Procedure	Count (%)
CT BRAIN WO	151,841 (22)
CT ABD/PELV W	78,205 (11)
CT CHEST WO	60,416 (9)
CT ABD/PELV WO	49,194 (7)
CT CAP W	40,446 (6)
CT CHEST PE	35,396 (5)
CT C‐SPINE WO	28,858 (4)
CT NECK W	25,091 (4)
CT CHEST W	19,366 (3)
CTA HEAD W	17,835 (3)

Abbreviation key: abdomen/pelvis (ABD/PELV), cervical spine (C‐SPINE), chest/abdomen/pelvis (CAP), computed tomography angiography (CTA), pulmonary embolism (PE), with contrast (W), without contrast (WO),

### 3.3 Monthly dose indices across procedures

Figure 1 and Figure 2 display the monthly median CTDI_vol_ and DLP, respectively, for the ten most utilized diagnostic procedures along with regression model‐derived trend lines. CTDI_vol_ is shown to decrease significantly for all ten procedures, with the largest percent decrease observed for CT CAP W (−5.03%/year). Monthly median DLP decreased significantly for seven of the procedures (CT ABD/PELV W, CT CHEST WO, CT ABD/PELV WO, CT CAP W, CT NECK W, CT CHEST W, and CTA HEAD W), with the largest percent decreases observed for CT ABD/PELV W (−8.05%/year) and CT CAP W (−9.7%/year). Patient weight and age were significant covariates (*p* < 0.05) in nearly all models for both CTDI_vol_ and DLP. The exception was age for CTDI_vol_ of CT C‐SPINE WO, for which the association with dose was not statistically significant (*p* = 0.30).

**FIGURE 1:**
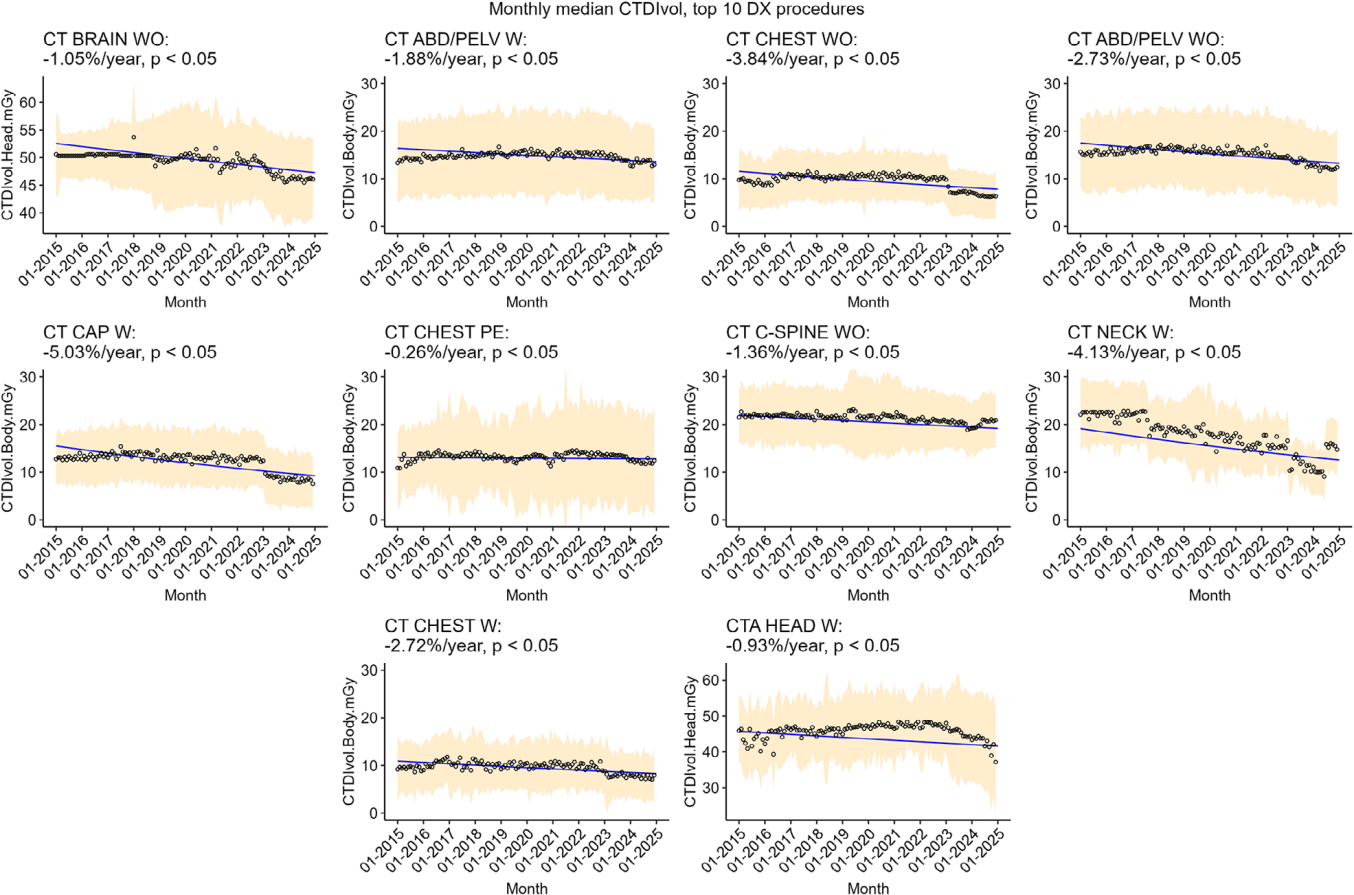
Scatter plots displaying the monthly median CTDI_vol_ (mGy) ± standard deviation (shaded region) for the 10 most‐used diagnostic procedures. Shown in blue is a linear regression trend line from a linear mixed effects model with time (as days since January 1, 2015), patient weight (kg), and patient age. Estimated dose trend (as % per year) and *p*‐value are shown below each procedure name.

**FIGURE 2:**
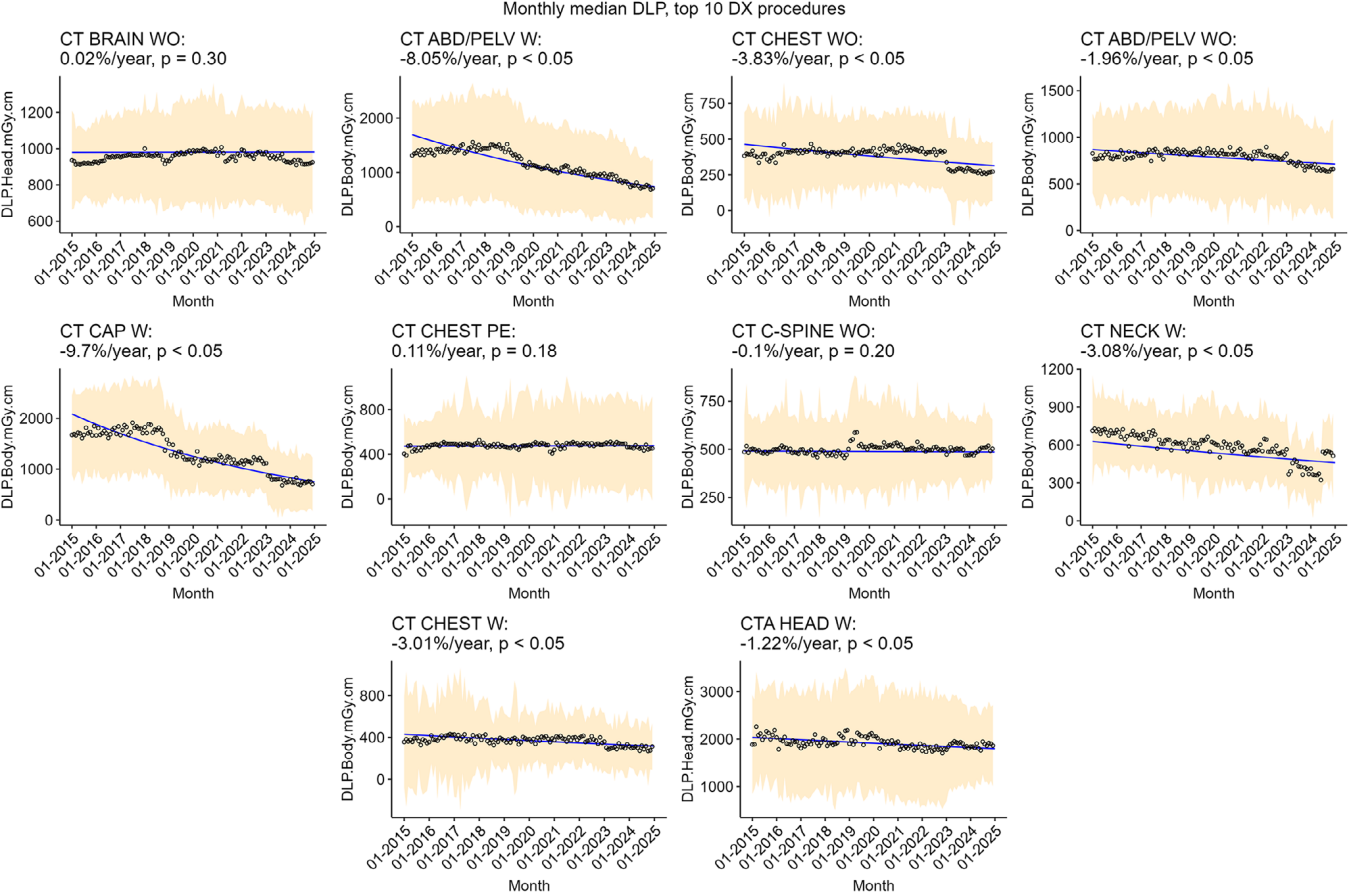
Scatter plots displaying the monthly median DLP (mGy×cm) ± standard deviation (shaded region) for the 10 most‐used diagnostic procedures. Shown in blue is a linear regression trend line from a linear mixed effects model with time (as days since January 1, 2015), patient weight (kg), and patient age. Estimated dose trend (as % per year) and *p*‐value are shown below each procedure name. Y‐axis limits are not standardized due to large differences in DLP standard deviation between procedures.

### 3.4 Quarterly dose indices across procedures and scanner models

The quarterly median CTDI_vol_ and DLP per scanner model are shown in Figures 3 and 4, respectively, for all ten procedures. For each procedure except CT NECK W, the lowest quarterly CTDI_vol_ and DLP are from the newer SOMATOM Drive and NAEOTOM Alpha models. Higher quarterly CTDI_vol_ and DLP are seen on the BrightSpeed, Brilliance 64, and Sensation 64 scanners, each older models that have since been decommissioned. Additionally, for CT ABD/PELV W and CT CAP W, a decrease in DLP is observed across all scanner models in the first quarter of 2019, which persists to the end of the observational window.

**FIGURE 3:**
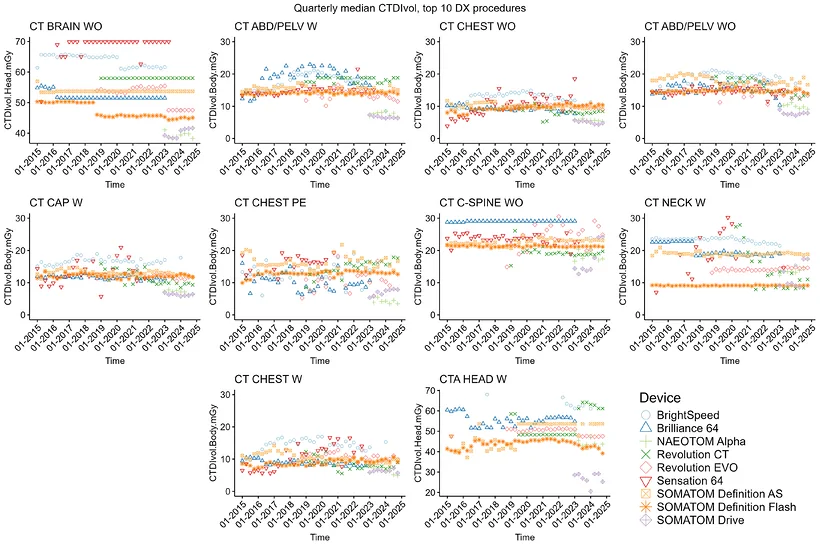
Scatter plots displaying the quarterly median CTDI_vol_ (mGy) per scanner model for the 10 most‐used diagnostic procedures.

**FIGURE 4:**
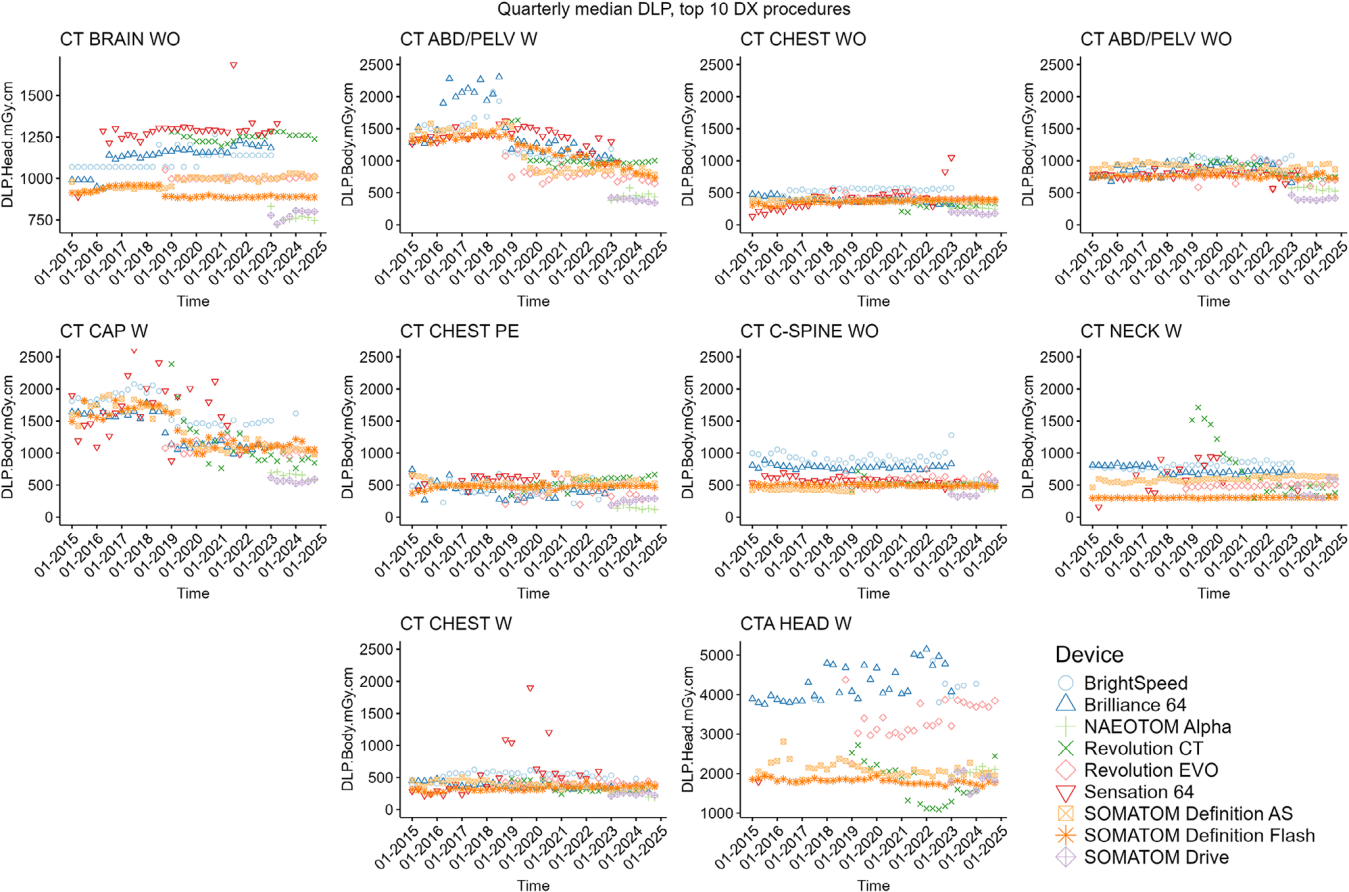
Scatter plots displaying the quarterly median DLP (mGy×cm) per scanner model for the 10 most‐used diagnostic procedures.

Sudden decreases in CTDI_vol_ for CT BRAIN WO and CT NECK W are observed on several scanner models, indicating protocol or technique changes (Figure 3). For CT BRAIN WO, such decreases occurred on the BrightSpeed, Revolution EVO, Definition Flash, and Brilliance 64. For CT NECK W, decreases occurred on the BrightSpeed and Brilliance 64.

### 3.5 Monthly mean acquisition count across procedures

For each procedure, the monthly mean acquisition count was calculated and is shown in Figure 5 along with Pearson correlation results. For non‐contrast procedures, CT CHEST WO showed a significant decrease in mean acquisition count (*r* = −0.2, *p* < 0.05) while CT BRAIN WO (*r* = 0.37, *p* < 0.05) and CT C‐SPINE WO (*r* = 0.57, *p* < 0.05) both showed significant increases. No change in mean acquisition count was observed for CT ABD/PELV WO. All six contrast procedures (CT ABD/PELV W, CT CAP W, CT CHEST PE, CT CHEST W, CT NECK W, CTA HEAD W) demonstrated significant (*p* < 0.05) decreases in monthly mean acquisition count, with CT ABD/PELV W (*r* = −0.94) and CT CAP W (*r* = −0.89) having the highest correlation coefficients.

**FIGURE 5:**
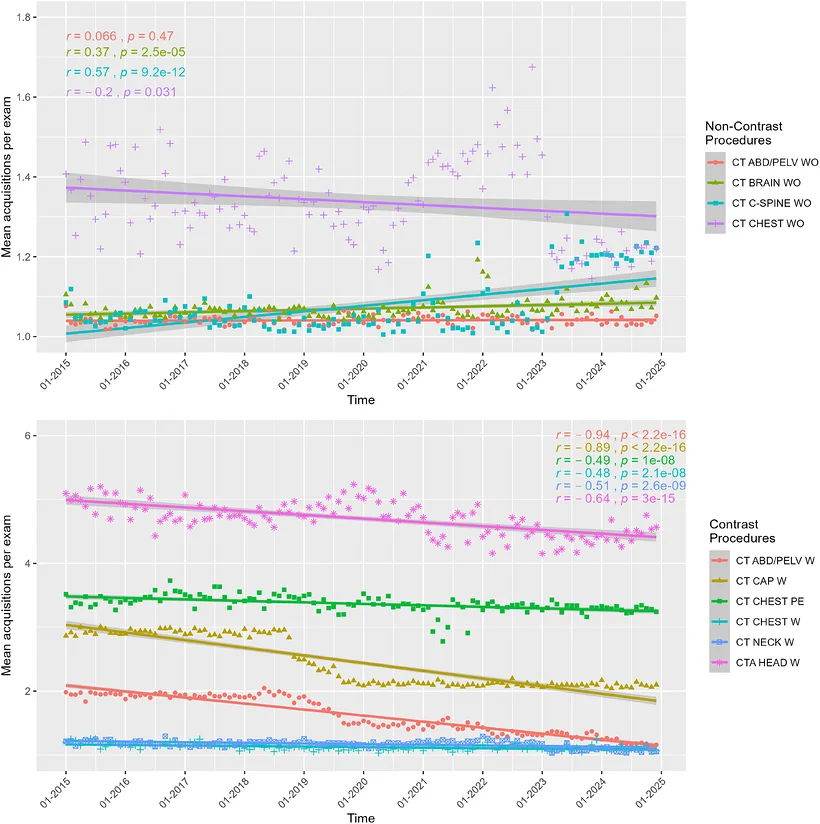
Plot displaying the mean number of acquisitions in a single exam for each month and non‐contrast (A, top) and contrast (B, bottom) procedure over the 10‐year period. For each procedure, linear trend lines are shown along with the Pearson correlation coefficient and *p*‐values, calculated between the monthly mean acquisition and time (as months since January 2015). Increases in mean number of acquisitions per exam for CT BRAIN WO and CT C‐SPINE WO were not corroborated by protocol changes or observed repeat imaging and may be due to a RDIM exam‐grouping software error.

### 3.6 Patient size

For each year from 2015–2024, mean patient weight in kilograms and mean patient diameter in millimeters are shown in Figure 6. The slope of the patient weight regression line was +0.10 kilograms per year (*r^2^* = 0.481, *p* = 0.026), while the slope of the patient diameter regression line was +1.01 mm/year (*r^2^* = 0.724, *p* = 0.002).

**FIGURE 6:**
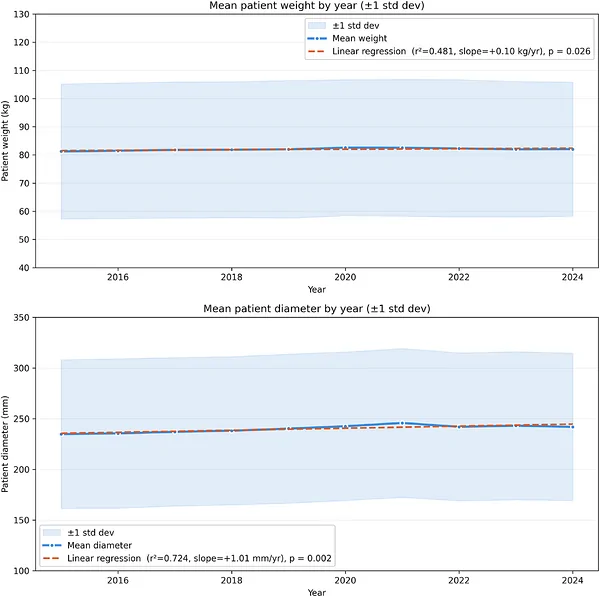
Yearly mean patient weight (top) and patient diameter (bottom) over the 10‐year period. Linear regression trendlines (orange dashed line) were fitted to the mean values of weight and diameter for each year.

### 3.7 Annual phantom image quality metrics

Annual ACR testing images were retrieved and processed with CTPro for three scanners in 2015 (SOMATOM Definition AS, SOMATOM Flash, and Sensation 64) and two scanners in 2024 (SOMATOM Drive, NAEOTOM Alpha). A summary of the acquisition parameters and CTPro ACR QC results are shown in Table 3. The CHO detectability index (d’), defined as the signal‐to‐noise ratio of the CHO test statistic,^27^, ^29^ is shown in Figure 7 with respect to CTDI_vol_ for each low contrast object. For all three test objects, d’ was higher on the Alpha in 2024 when compared to the 2015 phantom tests, despite lower CTDI_vol_. The Drive also exhibited higher d’ in 2024 for the 4 and 6 mm objects. Additionally, the maximum visible bar pattern (measured using Module 4) was 7 line pairs per cm for both the Drive and Alpha, compared to 6 line pairs per cm for the Definition AS, Flash, and Sensation 64.

**TABLE 3:** Summary of acquisition parameters, reconstruction parameters, and CTPro ACR QC results for Modules 2–4, including noise magnitude, contrast‐to‐noise ratio (CNR), detectability index (d’) for 4 mm, 5 mm, and 6 mm test objects, average noise power spectrum spatial frequency (favg), and the maximum visible bar pattern.

	Model	Definition Flash	Definition AS	Sensation 64	Drive	Alpha
	**Year of ACR Testing**	2015	2015	2015	2024	2024
	**Kernel**	I40f (2)	I40f (2)	B40f	Br40f (3)	Br44f (2)
	**Recon FOV (mm)**	220	215	220	220	220
	**Rotation Time (ms)**	500	500	500	500	312
	**Pitch**	0.6	0.6	0.8	0.6	0.8
	**Eff. mAs**	126	114	192	88.5	129.7
	**CTDI_vol_ (mGy)**	14.2	13.9	18.3	9.4	10.3
**Module 2**	**Noise (HU)**	4.13	4.82	5.25	4.5	4.82
	**CNR**	0.59	1.37	1.27	1.37	1.43
	**d' (4 mm)**	0.43 ± 0.253	0.64 ± 0.249	0.34 ± 0.228	0.72 ± 0.412	0.70 ± 0.323
	**d' (5 mm)**	0.58 ± 0.313	0.99 ± 0.394	0.37 ± 0.260	0.87 ± 0.427	1.89 ± 0.466
	**d' (6 mm)**	0.92 ± 0.333	1.31 ± 0.346	0.51 ± 0.289	3.22 ± 0.829	2.11 ± 0.524
**Module 3**	***f*_avg_ (cm^−1^)**	2.68	2.79	3.06	2.75	2.97
	**Noise magnitude (HU)**	4.21	4.5	5.71	4.36	4.79
**Module 4**	**Max visible bar pattern (lp/cm)**	6	6	6	7	7

All acquisitions were at 120 kVp and reconstructed with 5 mm slice thickness and 5 mm slice interval.

**FIGURE 7:**
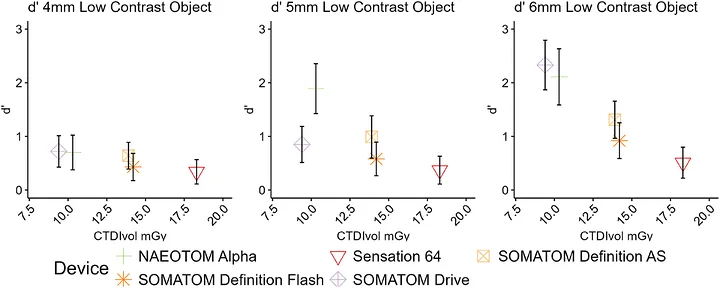
Detectability index (d’) and CTDI_vol_ for each low contrast object in Module 2 of the ACR phantom, scanned in 2015 (SOMATOM Definition AS, SOMATOM Definition Flash, Sensation 64) and 2024 (SOMATOM Drive and NAEOTOM Alpha). Reconstruction kernels were I40f (Flash, AS), B40f (Sensation 64), Br40f (Drive), and Br44f (Alpha). All scans were at 120 kVp and reconstructed with 5 mm slice thickness and 5 mm slice interval.  Comparisons are intended as contextual as protocols are not fully matched.

## 4 DISCUSSION

Of the ten most utilized diagnostic procedures, decreases in CTDI_vol_ over the 10‐year period (Figure 1) were observed for all procedures, while decreases in DLP (Figure 2) were observed in all but three procedures (CT BRAIN WO, CT CHEST PE, and CT C‐SPINE). These aggregate trends were further investigated at the scanner model level in Figure 3 (CTDI_vol_) and Figure 4 (DLP), where both scanner model and institutional inflection points can be observed. For most procedures, reductions in CTDI_vol_ and DLP are observed beginning in the first quarter of 2023, corresponding with the addition of five newer‐model scanners to the fleet in 2023 and 2024 (one Siemens NAEOTOM Alpha and four Siemens SOMATOM Drive, Table 1) following the removal of a Siemens Sensation 64, a GE BrightSpeed, and a Philips Brilliance 64.

These dose reductions between new and older‐model CT scanners are expected, due to the utilization of advanced hardware and dose reduction techniques developed by all major manufacturers. For example, the Siemens SOMATOM Drive features an upgraded detector (Stellar Infinity) with improved dose efficiency due to integrated electronics,^30^, ^31^ as well as the addition of optional tin filters to preferentially filter low‐energy components of the x‐ray beams in low‐dose CT CHEST WO procedures.^32^, ^33^

While some form of dose modulation (i.e. automatic exposure control) was present on the older scanners, it is expected that updated algorithms on newer scanners can further minimize dose. On the Siemens SOMATOM Drive, protocols for the ten most utilized procedures all utilized automated tube current modulation (CARE Dose 4D), while nine of ten (excluding CTA HEAD W) utilized dose‐optimized tube voltage selection (CARE kV).

Lastly, the use of iterative reconstruction techniques can provide appreciable dose reductions by reducing image noise compared to filtered back projection while maintaining comparable image quality and resolution.^34^ Older scanner models may either have no iterative reconstruction capabilities or 2^nd^ generation algorithms, while our current protocols on the SOMATOM Drive utilize 3^rd^ generation iterative reconstruction techniques on all ten of the most utilized procedures.

While the impact of new scanner models on dose indices can be observed across all procedures, procedure‐specific changes also occurred over the 10‐year period. A noticeable stepwise DLP decrease is observed with CT ABD/PELV W (11% of all exams) and CT CAP W (6% of all exams) in the first quarter of 2019 (Figure 2, Figure 4). This change is primarily due to the elimination of the delayed phase acquisitions in abdomen and pelvic contrast exams^35^ and can also be seen in the monthly mean acquisitions per exam for the two procedures (Figure 5).

Additionally, multiple stepwise reductions in CTDI_vol_ (Figure 3) for CT BRAIN WO and CT NECK W occurred on several scanner models, including the BrightSpeed, Revolution EVO, Definition Flash, and Brilliance 64 for CT BRAIN WO, and BrightSpeed and Brilliance 64 for CT NECK W. For each scanner, clinical images and protocol documentation were reviewed to determine the cause of the sudden decrease. The decreases in CTDI_vol_ for CT NECK W on the Brilliance 64 and BrightSpeed both occurred due to a change from a fixed technique to enabling automatic exposure control, although it should be highlighted that the BrightSpeed decrease was small. For CT BRAIN WO procedures, protocol changes from axial to helical scanning with lower target doses were implemented in 2016 for the Brilliance 64 and 2023 for the Revolution EVO. In mid‐2018, the routine CT BRAIN WO protocol on the Definition Flash was modified to use dual energy rather than single energy, and the target dose was lowered. On the BrightSpeed, no known protocol changes were made prior to the observed median CTDI_vol_ decrease (early 2020), however, a software upgrade was performed around this time and may have contributed.

Despite the reductions in CTDI_vol_ for CT BRAIN WO (−1.05%/yr), CT CHEST PE (−0.26%/yr), and CT C‐SPINE WO (−1.36%/yr) (Figure 1), DLP remained unchanged (Figure 2) and monthly mean acquisitions per exam for CT BRAIN WO and CT C‐SPINE WO significantly increased during the 10‐year period (Figure 5), implying an increase in additional or repeat acquisitions unsupported by protocol changes. An investigation into exams with high acquisition counts for these procedures revealed that for some exams, Radimetrics was unable to perform a DICOM Query/Retrieve (Q/R), resulting in some orders with multiple procedures to be incorrectly grouped under the first procedure. For CT BRAIN WO, some acquisition counts included subsequent contrast exams; for CT C‐SPINE WO, subsequent lumbar and thoracic procedures were erroneously included. These additional acquisitions would artifactually increase total examination scan length and subsequently DLP. At present there is no method in Radimetrics to filter exams where the Q/R process failed, and manual review of the large dataset is infeasible.

Due to the positive relationship between patient size and CTDI_vol_ and DLP, it is possible for increases in average patient size over time to result in corresponding increases in radiation output. Despite not observing any steady increases in CTDI_vol_ or DLP across procedures, and accounting for patient weight in multivariable linear regression, we report average patient weight (kg) and average patient diameter (mm, measured by Radimetrics) for each year in Figure 6. Linear regression analysis for both measurements reveal small year over year increases for both, with slopes of +0.10 kg and +1.01 mm per year. While both increases are statistically significant, the increased radiation output would likely be negligible given the small overall increase in patient size.

It is well understood that CTDI_vol_ and DLP, as measures of *radiation output*, should not be used for *individual dose estimation* as they are not size‐specific.^36^ It has been proposed that CTDI_vol_, along with a size‐dependent conversion factor, provides an estimate of average absorbed dose referred to as the size‐specific dose estimate (SSDE).^24^, ^37^ Recent works on CT dose effects and optimization often utilize effective dose, estimated using organ weighting factors from the International Commission on Radiological Protection (ICRP) Publication 103.^38^ However, calculations of effective dose are subject to inherent uncertainties, do not account for all individual variations, and utilize estimates of relative biologic risk, which have changed over time and may continue to evolve.^39^, ^40^

In this work, we focused on CTDI_vol_ and DLP as operational dose indices, as the patient size data required for consistent SSDE calculation were incompletely available and, in some cases inconsistent in the RDIM software for the observational period. While CTDI_vol_ and DLP do not measure dose to the patient, institutional dose management programs and regulatory benchmarks are still commonly expressed in these dose indices and they remain meaningful operational metrics for tracking changes over time.

As decreases in dose may be accompanied by undesired decreases in image quality and thus diagnostic value, a retrospective evaluation of image quality was performed using historical ACR annual testing images acquired in 2015 and 2024. Acquisition and reconstruction parameters differed between scanners (Table 3); therefore, the phantom analysis was used as a retrospective qualitative and semi‐quantitative contextual assessment of low contrast performance rather than a controlled comparison. Analysis of these images using CTPro (https://ctpro.net/home) demonstrated low contrast detectability improvements on scanners used in 2024 compared to those used in 2015 (Figure 7), with one exception noted for the 5 mm test object on the SOMATOM Drive. This may be explained by a limitation of the retrospective analysis: recommended methods for CHO calculation include at least three repeated scans and 0.5 mm slice thickness to minimize bias, conditions that were not met by using historical data consisting of one scan and 5 mm slices.^27^ Furthermore, high‐contrast resolution analysis using Module 4 of the phantom shows an increase in the maximum visible bar pattern, from 6 line pairs per cm (Definition Flash, Definition AS, Sensation 64) to 7 line pairs per cm (Drive, Alpha), demonstrating spatial resolution improvements on newer scanners.

Dose optimization is a collaborative effort between medical physicists and radiologists. Therefore, it was assumed that all decreases in dose due to enabling or disabling automatic exposure control, reducing target doses, or any other technique changes would be reverted should acceptable diagnostic quality fail to be maintained. During the 10‐year period, one observable case of a reversed change occurred. In mid‐2023, the monthly median CTDI_vol_ for CT C‐SPINE WO decreased due to the increasing volume of exams on three new SOMATOM Drive scanners with lower dose protocols (Figure 1, Figure 3). By 2024, dose had increased as the protocol was modified to improve image quality at the request of our radiologists.

An additional limitation arises from the decision to group exams by procedure rather than protocol. As procedures are less specific than protocols, it is possible for two protocols to fall under the same procedure despite being used for different conditions and thus have different acquisition parameters and dose output. However, the large number of unique procedure names in the dataset (1,832) would make harmonization laborious and difficult, and introduces the problem of handling protocols changing names while on the same scanner, or attempting to match similar protocols with different names on different scanners.

Lastly, the multivariable linear regression models used to investigate dose indices over time did not include scanner model as a covariate. As scanner models were introduced and decommissioned during the study period and do not fully overlap one another (e.g., the BrightSpeed and SOMATOM Drive were never in use at the same time), time and scanner model are confounded. A mixed‐effects model with scanner model‐specific random slopes was considered but was rejected as the underlying confounding factors would still be present. Future work on an expanded time scale could focus on periods without significant scanner fleet changes and employ segmented regression or interrupted time series analysis to better quantify the contribution of specific interventions to reduce dose.

Our analysis of CT dose indices over a 10‐year period demonstrates a significant decrease in per‐exam dose for the majority of routine procedures performed in a large health system. These decreases, due to a combination of technology upgrades and technique changes, highlight adherence to the ALARA principle. This is especially critical in a time when the relationship between CT exams and future cancer risk is not fully understood, particularly due to the difficulties of accounting for reverse causation and confounding indications in epidemiological work.^41^, ^42^ These uncertainties underscore the importance of maintaining the complex balance of image quality and dose, and leveraging new techniques and technologies. Automated tube current modulation, increased detector efficiency, advanced reconstruction algorithms, and enhanced protocol flexibility and workflow tools can facilitate application‐ and indication‐specific protocol optimization that ultimately benefit patients.

## 5 CONCLUSIONS

A retrospective review of 695,575 CT exams over a 10‐year period (2015–2024) was conducted to evaluate CTDI_vol_ and DLP trends over time. Both dose indices decreased across multiple high‐utilization procedures and can be attributed to protocols on new scanners with advanced dose reduction capabilities, a decrease in multiphase and repeat imaging, and the ongoing efforts of medical physicists, technologists, and radiologists to balance dose and image quality.

## AUTHOR CONTRIBUTIONS

MPS and JMW conceived the research project. JMW organized and analyzed the data and performed the statistical analyses. AA, NEM, and MPS assisted with study design, data organization, and analysis. AA, NEM, MPS, and DRL provided clinical expertise and review of the manuscript. JMW drafted the preliminary manuscript and all co‐authors revised and approved the final manuscript.

## CONFLICT OF INTEREST SATEMENT

Mark P. Supanich has received speaking fees from Bayer. There are no additional activities to disclose.

## ETHICS STATEMENT

This retrospective study received approval from the Rush Institutional Review Board (ID# 16062204‐IRB01) with waiver of written informed consent.

## FUNDING INFORMATION

This research received no external financial support.

## Supporting information




Supporting Information contains the procedure mapping dictionary used to harmonize imaging procedure names across multiple sites and scanners over the ten year period.

## Data Availability

The data that support the findings of this study are available upon reasonable request made to the corresponding author.
