# Supplementary material for: Institutional CT dose reduction: A retrospective evaluation of dose indices from 2015–2024
Source: J Appl Clin Med Phys. 2026 Oct 5;27(10):e70823. doi: 10.1002/acm2.70823 (PMC13639238; doi:10.1002/acm2.70823)
Supplement: Supplementary file 1 [file ACM2-27-e70823-s001.docx]

**Table S-1:** Manually created dictionary to map procedure names exported from Radimetrics to generic procedure names, to account for procedure name variation.

| **Generic** | **Procedures (from Radimetrics/DICOM)** |
| --- | --- |
| CT ABD W | CT ABDOMEN W IV CONT |
| CT ABD W | CT ABDOMEN WITH |
| CT ABD W | CT ABDOMEN WITH IV CONTRAST |
| CT ABD W | CT ABDOMEN WITH IV CONTRAST,MM Reading |
| CT ABD W and WO | CT ABDOMEN WITHOUT AND WITH IV CONTRAST |
| CT ABD W and WO | CT ABDOMEN WITHOUT AND WITH IV CONTRAST,CT ABDOMEN WITHOUT AND WITH IV CONTRAST |
| CT ABD W and WO | CT ABDOMEN WITHOUT AND WITH IV CONTRAST,CT Dual Energy |
| CT ABD W and WO | CT ABDOMEN WITHOUT AND WITH IV CONTRAST,MM Reading |
| CT ABD W and WO | CT ABDOMEN WO AND W IV CONT |
| CT ABD WO | CT ABDOMEN WITHO |
| CT ABD WO | CT ABDOMEN WITHOUT IV CONTRAST |
| CT ABD WO | CT ABDOMEN WITHOUT IV CONTRAST,CT ABDOMEN PELVIS WITHOUT IV CONTRAST |
| CT ABD WO | CT ABDOMEN WITHOUT IV CONTRAST,CT ABDOMEN WITHOUT IV CONTRAST |
| CT ABD WO | CT ABDOMEN WITHOUT IV CONTRAST,CT ABDOMEN WITHOUT IV CONTRAST,MM Reading |
| CT ABD WO | CT ABDOMEN WITHOUT IV CONTRAST,CT Dual Energy |
| CT ABD WO | CT ABDOMEN WITHOUT IV CONTRAST,MM Reading |
| CT ABD WO | CT ABDOMEN WO IV CONT |
| CT ABD/PELV W | CT ABDOMEN PELVI |
| CT ABD/PELV W | CT ABDOMEN PELVI,CT ABDOMEN PELVI |
| CT ABD/PELV W | CT ABDOMEN PELVIS WITH IV CONTRAST |
| CT ABD/PELV W | CT ABDOMEN PELVIS WITH IV CONTRAST,CT ABDOMEN PELVIS WITH IV CONTRAST |
| CT ABD/PELV W | CT ABDOMEN PELVIS WITH IV CONTRAST,CT ABDOMEN PELVIS WITH IV CONTRAST,CT ABDOMEN PELVIS WITH IV CONTRAST |
| CT ABD/PELV W | CT ABDOMEN PELVIS WITH IV CONTRAST,CT ABDOMEN PELVIS WITH IV CONTRAST,CT ABDOMEN PELVIS WITH IV CONTRAST,CT ABDOMEN PELVIS WITH IV CONTRAST |
| CT ABD/PELV W | CT ABDOMEN PELVIS WITH IV CONTRAST,CT ABDOMEN PELVIS WITH IV CONTRAST,CT ABDOMEN PELVIS WITH IV CONTRAST,CT ABDOMEN PELVIS WITH IV CONTRAST,CT ABDOMEN PELVIS WITH IV CONTRAST |
| CT ABD/PELV W | CT ABDOMEN PELVIS WITH IV CONTRAST,CT ABDOMEN PELVIS WITH IV CONTRAST,CT ABDOMEN PELVIS WITH IV CONTRAST,CT ANGIOGRAM CHEST, ABDOMEN, AND PELVIS WITH IV CONTRAST |
| CT ABD/PELV W | CT ABDOMEN PELVIS WITH IV CONTRAST,CT ABDOMEN PELVIS WITH IV CONTRAST,CT ABDOMEN PELVIS WITH IV CONTRAST,CT Dual Energy |
| CT ABD/PELV W | CT ABDOMEN PELVIS WITH IV CONTRAST,CT ABDOMEN PELVIS WITH IV CONTRAST,CT Dual Energy |
| CT ABD/PELV W | CT ABDOMEN PELVIS WITH IV CONTRAST,CT ABDOMEN PELVIS WITH IV CONTRAST,CT Dual Energy,CT ABDOMEN PELVIS WITH IV CONTRAST |
| CT ABD/PELV W | CT ABDOMEN PELVIS WITH IV CONTRAST,CT ABDOMEN PELVIS WITH IV CONTRAST,MM Reading |
| CT ABD/PELV W | CT ABDOMEN PELVIS WITH IV CONTRAST,CT ABDOMEN PELVIS WITHOUT AND WITH IV CONTRAST |
| CT ABD/PELV W | CT ABDOMEN PELVIS WITH IV CONTRAST,CT ABDOMEN PELVIS WITHOUT IV CONTRAST,CT Dual Energy |
| CT ABD/PELV W | CT ABDOMEN PELVIS WITH IV CONTRAST,CT Dual Energy |
| CT ABD/PELV W | CT ABDOMEN PELVIS WITH IV CONTRAST,CT Dual Energy,CT ABDOMEN PELVIS WITH IV CONTRAST |
| CT ABD/PELV W | CT ABDOMEN PELVIS WITH IV CONTRAST,MM Reading |
| CT ABD/PELV W and WO | CT ABDOMEN PELVIS WITHOUT AND WITH IV CONTRAST |
| CT ABD/PELV W and WO | CT ABDOMEN PELVIS WITHOUT AND WITH IV CONTRAST,CT ABDOMEN PELVIS WITHOUT AND WITH IV CONTRAST |
| CT ABD/PELV W and WO | CT ABDOMEN PELVIS WITHOUT AND WITH IV CONTRAST,CT ABDOMEN PELVIS WITHOUT AND WITH IV CONTRAST,MM Reading |
| CT ABD/PELV W and WO | CT ABDOMEN PELVIS WITHOUT AND WITH IV CONTRAST,CT Dual Energy |
| CT ABD/PELV W and WO | CT ABDOMEN PELVIS WITHOUT AND WITH IV CONTRAST,MM Reading |
| CT ABD/PELV W and WO | CT ABDOMEN PELVIS WITHOUT AND WITH IV CONTRAST,MM Reading,CT ABDOMEN PELVIS WITHOUT AND WITH IV CONTRAST |
| CT ABD/PELV W and WO | CT ABDOMEN PELVIS WO AND W IV CONT |
| CT ABD/PELV WO | CT ABDOMEN PELVIS WITHOUT IV CONTRAST |
| CT ABD/PELV WO | CT ABDOMEN PELVIS WITHOUT IV CONTRAST,CT ABDOMEN PELVIS WITHOUT IV CONTRAST |
| CT ABD/PELV WO | CT ABDOMEN PELVIS WITHOUT IV CONTRAST,CT ABDOMEN PELVIS WITHOUT IV CONTRAST,CT ABDOMEN PELVIS WITHOUT IV CONTRAST |
| CT ABD/PELV WO | CT ABDOMEN PELVIS WITHOUT IV CONTRAST,CT ABDOMEN PELVIS WITHOUT IV CONTRAST,CT ABDOMEN PELVIS WITHOUT IV CONTRAST,CT ABDOMEN PELVIS WITHOUT IV CONTRAST |
| CT ABD/PELV WO | CT ABDOMEN PELVIS WITHOUT IV CONTRAST,CT ABDOMEN PELVIS WITHOUT IV CONTRAST,CT ABDOMEN PELVIS WITHOUT IV CONTRAST,CT ABDOMEN PELVIS WITHOUT IV CONTRAST,CT ABDOMEN PELVIS WITHOUT IV CONTRAST,CT ABDOMEN PELVIS WITHOUT IV CONTRAST |
| CT ABD/PELV WO | CT ABDOMEN PELVIS WITHOUT IV CONTRAST,CT ABDOMEN PELVIS WITHOUT IV CONTRAST,CT Dual Energy |
| CT ABD/PELV WO | CT ABDOMEN PELVIS WITHOUT IV CONTRAST,CT ABDOMEN PELVIS WITHOUT IV CONTRAST,MM Reading |
| CT ABD/PELV WO | CT ABDOMEN PELVIS WITHOUT IV CONTRAST,CT Dual Energy |
| CT ABD/PELV WO | CT ABDOMEN PELVIS WITHOUT IV CONTRAST,MM Reading |
| CT ABD/PELV WO | CT ABDOMEN PELVIS WITHOUT IV CONTRAST,MM Reading,None |
| CT ABD/PELV WO | CT ABDOMEN PELVIS WO IV CONT |
| CT ABD/PELV WO | CT ABDOMEN PELVIS WO IV CONT,CT ABDOMEN PELVIS WO IV CONT |
| CT ANKLE W | CT ANKLE (HINDFO |
| CT ANKLE W | CT ANKLE (HINDFOOT) LEFT WITH IV CONTRAST |
| CT ANKLE W | CT ANKLE (HINDFOOT) LEFT WITHOUT IV CONTRAST,CT ANKLE (HINDFOOT) LEFT WITHOUT IV CONTRAST |
| CT ANKLE W | CT ANKLE (HINDFOOT) LEFT WITHOUT IV CONTRAST,CT Dual Energy |
| CT ANKLE W | CT ANKLE (HINDFOOT) RIGHT WITH IV CONTRAST |
| CT ANKLE W | CT ANKLE (HINDFOOT) RIGHT WITHOUT IV CONTRAST,CT ANKLE (HINDFOOT) RIGHT WITHOUT IV CONTRAST |
| CT ANKLE W and WO | CT ANKLE (HINDFOOT) LEFT WITHOUT AND WITH IV CONTRAST |
| CT ANKLE W and WO | CT ANKLE (HINDFOOT) RIGHT WITHOUT AND WITH IV CONTRAST |
| CT ANKLE WO | CT ANKLE (HINDFOOT) LEFT WITHOUT IV CONTRAST |
| CT ANKLE WO | CT ANKLE (HINDFOOT) RIGHT WITHOUT IV CONTRAST |
| CT ANKLE WO | CT LT ANKLE (HINDFOOT) WITHOUT IV CONTRAST |
| CT ANKLE WO | CT RT ANKLE (HINDFOOT) WITHOUT IV CONTRAST |
| CT BILATERAL TMJS WO | CT BILATERAL TMJS WITHOUT IV CONTRAST |
| CT BILATERAL TMJS WO | CT BILATERAL TMJs WITHOUT IV CONTRAST |
| CT BILATERAL TMJS WO | CT TMJS BILATERAL WITH IV CONTRAST |
| CT BRACHIAL PLEXUS RIGHT WITH IV CONTRAST | CT BRACHIAL PLEXUS RIGHT WITH IV CONTRAST |
| CT BRAIN MRGFUS | CT BRAIN FOR MRGFUS PLANNING |
| CT BRAIN MRGFUS | CT BRAIN FOR MRgFUS PLANNING |
| CT BRAIN MRGFUS | CT BRAIN WITHOUT IV CONTRAST FOR MRGFUS PLANNING |
| CT BRAIN PERF | CT BRAIN PERFUSION |
| CT BRAIN PERF | CT BRAIN PERFUSION WITH DIAMOX |
| CT BRAIN PERF | CT BRAIN PERFUSION,CT BRAIN PERFUSION |
| CT BRAIN PERF | CT BRAIN WITH IV CONTRAST PERFUSION WITH DIAMOX |
| CT BRAIN PERF | CT BRAIN WITH IV CONTRAST PERFUSION WITH DIAMOX,CT Dual Energy |
| CT BRAIN PERF | CT PERFUSION BRAIN W IV CONT |
| CT BRAIN PERF | CT PERFUSION WITH CONTRAST CBF |
| CT BRAIN PERF | CT PERFUSION WITH CONTRAST CBF,CT PERFUSION WITH CONTRAST CBF |
| CT BRAIN PERF | CT PERFUSION WITH CONTRAST CBF,CT PERFUSION WITH CONTRAST CBF,CT PERFUSION WITH CONTRAST CBF |
| CT BRAIN PERF | CT PERFUSION WITH IV CONTRAST CBF,CT PERFUSION WITH IV CONTRAST CBF,CT Dual Energy |
| CT BRAIN PERF | CT PERFUSION WITH IV CONTRAST CBF,MM Reading |
| CT BRAIN STROKE | CT BRAIN STROKE |
| CT BRAIN STROKE | CT BRAIN STROKE PROTOCOL |
| CT BRAIN STROKE | CT BRAIN STROKE PROTOCOL,CT BRAIN STROKE PROTOCOL |
| CT BRAIN STROKE | CT BRAIN STROKE TRIAL |
| CT BRAIN STROKE | CT BRAIN WITHOUT IV CONTRAST STROKE PROTOCOL |
| CT BRAIN STROKE | CT BRAIN WITHOUT IV CONTRAST STROKE PROTOCOL,CT BRAIN WITHOUT IV CONTRAST STROKE PROTOCOL |
| CT BRAIN STROKE | CT BRAIN WITHOUT IV CONTRAST STROKE PROTOCOL,CT Dual Energy |
| CT BRAIN STROKE | CT BRAIN WITHOUT IV CONTRAST STROKE PROTOCOL,MM Reading |
| CT BRAIN STROKE | CT BRAIN WITHOUT IV CONTRAST,CT BRAIN STROKE PROTOCOL |
| CT BRAIN W | CT BRAIN W IV CONT |
| CT BRAIN W | CT BRAIN WITH IV CONTRAST |
| CT BRAIN W | CT BRAIN WITH IV CONTRAST,MM Reading |
| CT BRAIN W and WO | CT BRAIN WITHOUT AND WITH IV CONTRAST |
| CT BRAIN W and WO | CT BRAIN WITHOUT AND WITH IV CONTRAST,CT BRAIN WITHOUT AND WITH IV CONTRAST |
| CT BRAIN W and WO | CT BRAIN WITHOUT AND WITH IV CONTRAST,CT Dual Energy |
| CT BRAIN W and WO | CT BRAIN WO AND W IV CONT |
| CT BRAIN WITH | CT BRAIN WITH IV CONTRAST,CT Dual Energy |
| CT BRAIN WO | CT BRAIN DUAL ENERGY WITHOUT IV CONTRAST |
| CT BRAIN WO | CT BRAIN WITHOUT |
| CT BRAIN WO | CT BRAIN WITHOUT IV CONTRAST |
| CT BRAIN WO | CT BRAIN WITHOUT IV CONTRAST,CT BRAIN WITHOUT IV CONTRAST |
| CT BRAIN WO | CT BRAIN WITHOUT IV CONTRAST,CT BRAIN WITHOUT IV CONTRAST,CT BRAIN WITHOUT IV CONTRAST |
| CT BRAIN WO | CT BRAIN WITHOUT IV CONTRAST,CT BRAIN WITHOUT IV CONTRAST,CT BRAIN WITHOUT IV CONTRAST,CT BRAIN WITHOUT IV CONTRAST |
| CT BRAIN WO | CT BRAIN WITHOUT IV CONTRAST,CT BRAIN WITHOUT IV CONTRAST,CT BRAIN WITHOUT IV CONTRAST,CT BRAIN WITHOUT IV CONTRAST,CT BRAIN WITHOUT IV CONTRAST |
| CT BRAIN WO | CT BRAIN WITHOUT IV CONTRAST,CT BRAIN WITHOUT IV CONTRAST,CT BRAIN WITHOUT IV CONTRAST,CT BRAIN WITHOUT IV CONTRAST,CT BRAIN WITHOUT IV CONTRAST,CT BRAIN WITHOUT IV CONTRAST |
| CT BRAIN WO | CT BRAIN WITHOUT IV CONTRAST,CT BRAIN WITHOUT IV CONTRAST,CT BRAIN WITHOUT IV CONTRAST,CT BRAIN WITHOUT IV CONTRAST,CT Dual Energy |
| CT BRAIN WO | CT BRAIN WITHOUT IV CONTRAST,CT BRAIN WITHOUT IV CONTRAST,CT BRAIN WITHOUT IV CONTRAST,CT Dual Energy |
| CT BRAIN WO | CT BRAIN WITHOUT IV CONTRAST,CT BRAIN WITHOUT IV CONTRAST,CT Dual Energy |
| CT BRAIN WO | CT BRAIN WITHOUT IV CONTRAST,CT BRAIN WITHOUT IV CONTRAST,MM Reading |
| CT BRAIN WO | CT BRAIN WITHOUT IV CONTRAST,CT Dual Energy |
| CT BRAIN WO | CT BRAIN WITHOUT IV CONTRAST,CT Dual Energy,CT BRAIN WITHOUT IV CONTRAST |
| CT BRAIN WO | CT BRAIN WITHOUT IV CONTRAST,MM Reading |
| CT BRAIN WO | CT BRAIN WITHOUT,CT BRAIN WITHOUT |
| CT CAP W | CT CHEST ABDOMEN PELVIS WITH IV CONTRAST |
| CT CAP W | CT CHEST, ABDOMEN & PELVIS WITH IV CONTRAST |
| CT CAP W | CT CHEST, ABDOMEN & PELVIS WITH IV CONTRAST,CT CHEST, ABDOMEN & PELVIS WITH IV CONTRAST |
| CT CAP W | CT CHEST, ABDOMEN & PELVIS WITH IV CONTRAST,CT CHEST, ABDOMEN & PELVIS WITH IV CONTRAST,CT CHEST, ABDOMEN & PELVIS WITH IV CONTRAST |
| CT CAP W | CT CHEST, ABDOMEN & PELVIS WITH IV CONTRAST,CT CHEST, ABDOMEN & PELVIS WITH IV CONTRAST,CT Dual Energy |
| CT CAP W | CT CHEST, ABDOMEN & PELVIS WITH IV CONTRAST,CT CHEST, ABDOMEN & PELVIS WITH IV CONTRAST,MM Reading |
| CT CAP W | CT CHEST, ABDOMEN & PELVIS WITH IV CONTRAST,CT Dual Energy |
| CT CAP W | CT CHEST, ABDOMEN & PELVIS WITH IV CONTRAST,MM Reading |
| CT CAP W and WO | CT CHEST ABDOMEN PELVIS WO AND W IV CONT |
| CT CAP W and WO | CT CHEST WITH & ABDOMEN/PELVIS WITHOUT AND WITH CONTRAST |
| CT CAP W and WO | CT CHEST WITH & ABDOMEN/PELVIS WITHOUT AND WITH CONTRAST,CT CHEST WITH & ABDOMEN/PELVIS WITHOUT AND WITH CONTRAST |
| CT CAP W and WO | CT CHEST WITH & ABDOMEN/PELVIS WITHOUT AND WITH CONTRAST,CT CHEST, ABDOMEN & PELVIS WITH IV CONTRAST |
| CT CAP W and WO | CT CHEST WITH & ABDOMEN/PELVIS WITHOUT AND WITH CONTRAST,CT Dual Energy |
| CT CAP W and WO | CT CHEST WITH & ABDOMEN/PELVIS WITHOUT AND WITH CONTRAST,MM Reading |
| CT CAP W and WO | CT CHEST WITH AND ABDOMEN-PELVIS WITHOUT AND WITH CONTRAST |
| CT CAP WO | CT CHEST ABDOMEN PELVIS WO IV CONT |
| CT CAP WO | CT CHEST, ABDOMEN & PELVIS WITHOUT IV CONTRAST |
| CT CAP WO | CT CHEST, ABDOMEN & PELVIS WITHOUT IV CONTRAST,CT CHEST, ABDOMEN & PELVIS WITHOUT IV CONTRAST |
| CT CAP WO | CT CHEST, ABDOMEN & PELVIS WITHOUT IV CONTRAST,CT CHEST, ABDOMEN & PELVIS WITHOUT IV CONTRAST,CT CHEST, ABDOMEN & PELVIS WITHOUT IV CONTRAST |
| CT CAP WO | CT CHEST, ABDOMEN & PELVIS WITHOUT IV CONTRAST,CT Dual Energy |
| CT CAP WO | CT CHEST, ABDOMEN & PELVIS WITHOUT IV CONTRAST,MM Reading |
| CT CARDIAC IMAGING OVERREAD | CT CARDIAC IMAGING OVERREAD |
| CT CARDIAC IMAGING OVERREAD | CT CARDIAC IMAGING OVERREAD,CT CaScoring |
| CT CARDIAC IMAGING OVERREAD | CT CARDIAC IMAGING OVERREAD,CT CaScoring,None |
| CT CARDIAC STRUCT CONGENITAL HEART DISEASE | CT CARDIAC STRUCT CONGENITAL HEART DISEASE |
| CT CATHETER DRAINAGE PERITONEAL-RETROPERITONEAL | CT CATHETER DRAINAGE PERITONEAL-RETROPERITONEAL |
| CT CHEST NAVIGATIONAL BRONCHOSCOPY | CT CHEST NAVIGATIONAL BRONCHOSCOPY |
| CT Chest Navigational Bronchoscopy Without Contrast | CT Chest Navigational Bronchoscopy Without Contrast |
| CT CHEST PE | CT CHEST FOR PE |
| CT CHEST PE | CT CHEST FOR PE W IV CONT |
| CT CHEST PE | CT CHEST FOR PE WITH IV CONTRAST |
| CT CHEST PE | CT CHEST FOR PE WITH IV CONTRAST,CT CHEST FOR PE WITH IV CONTRAST |
| CT CHEST PE | CT CHEST FOR PE,CT CHEST FOR PE |
| CT CHEST SCREEN | CT CHEST FOLLOW-UP LOW DOSE |
| CT CHEST SCREEN | CT CHEST SCREENING |
| CT CHEST SCREEN | CT CHEST SCREENING - SELF PAY |
| CT CHEST SCREEN | CT CHEST SCREENING FOLLOW UP |
| CT CHEST SCREEN | CT CHEST SCREENING WITHOUT IV CONTRAST |
| CT CHEST SCREEN | CT CHEST SCREENING WITHOUT IV CONTRAST - SELF PAY |
| CT CHEST SCREEN | CT CHEST SCREENING WITHOUT IV CONTRAST,CT Dual Energy |
| CT CHEST SCREEN | CT CHEST WITHOUT IV CONTRAST FOLLOW-UP LOW DOSE |
| CT CHEST W | CT CHEST W IV CONT |
| CT CHEST W | CT CHEST WITH & |
| CT CHEST W | CT CHEST WITH IV |
| CT CHEST W | CT CHEST WITH IV CONTRAST |
| CT CHEST W | CT CHEST WITH IV CONTRAST,CT ANGIOGRAM CHEST FOR PE WITH IV CONTRAST |
| CT CHEST W | CT CHEST WITH IV CONTRAST,CT CHEST WITH IV CONTRAST |
| CT CHEST W | CT CHEST WITH IV CONTRAST,CT CHEST WITH IV CONTRAST,CT Dual Energy,CT CHEST WITH IV CONTRAST |
| CT CHEST W | CT CHEST WITH IV CONTRAST,CT Dual Energy |
| CT CHEST W | CT CHEST WITH IV CONTRAST,MM Reading |
| CT CHEST W and WO | CT CHEST WITHOUT AND WITH IV CONTRAST |
| CT CHEST W and WO | CT CHEST WO AND W IV CONT |
| CT CHEST WO | CT CHEST WITHOUT |
| CT CHEST WO | CT CHEST WITHOUT IV CONTRAST |
| CT CHEST WO | CT CHEST WITHOUT IV CONTRAST,CT CHEST WITH IV CONTRAST |
| CT CHEST WO | CT CHEST WITHOUT IV CONTRAST,CT CHEST WITHOUT IV CONTRAST |
| CT CHEST WO | CT CHEST WITHOUT IV CONTRAST,CT CHEST WITHOUT IV CONTRAST,CT CHEST WITHOUT IV CONTRAST |
| CT CHEST WO | CT CHEST WITHOUT IV CONTRAST,CT CHEST WITHOUT IV CONTRAST,MM Reading |
| CT CHEST WO | CT CHEST WITHOUT IV CONTRAST,CT Dual Energy |
| CT CHEST WO | CT CHEST WITHOUT IV CONTRAST,MM Reading |
| CT CHEST ZEPHYR PROTOCOL | CT CHEST ZEPHYR PROTOCOL |
| CT CHEST/ABD W | CT CHEST & ABDOM |
| CT CHEST/ABD W | CT CHEST & ABDOMEN WITH IV CONTRAST |
| CT CHEST/ABD W | CT CHEST ABDOMEN W IV CONT |
| CT CHEST/ABD W | CT CHEST, ABDOME |
| CT CHEST/ABD W | CT CHEST, ABDOME,CT CHEST, ABDOME |
| CT CHEST/ABD W and WO | CT CHEST WITH & ABDOMEN WITHOUT AND WITH IV CONTRAST |
| CT CHEST/ABD W and WO | CT CHEST WITH AND ABDOMEN WITHOUT AND WITH IV CONTRAST |
| CT CHEST/ABD W and WO | CT CHEST WITH AND CT ABDOMEN WITHOUT AND WITH IV CONTRAST |
| CT CHEST/ABD WO | CT CHEST & ABDOMEN WITHOUT IV CONTRAST |
| CT CHEST/ABD WO | CT CHEST & ABDOMEN WITHOUT IV CONTRAST,CT Dual Energy |
| CT CHEST/ABD WO | CT CHEST ABDOMEN WO IV CONT |
| CT CLAV W | CT CLAVICLE LEFT WITH IV CONTRAST |
| CT CLAV W | CT CLAVICLE RIGHT WITH IV CONTRAST |
| CT CLAV W and WO | CT CLAVICLE LEFT WITHOUT AND WITH IV CONTRAST |
| CT CLAV WO | CT CLAVICLE LEFT WITHOUT IV CONTRAST |
| CT CLAV WO | CT CLAVICLE RIGHT WITHOUT IV CONTRAST |
| CT COLON SCREEN | CT COLONOGRAPHY SCREENING |
| CT COLON W | CT COLONGRAPHY W IV CONT |
| CT COLON W | CT COLONGRAPHY WITH IV CONTRAST |
| CT COLON W | CT COLONGRAPHY WITH IV CONTRAST,CT COLONGRAPHY WITH IV CONTRAST,CT COLONGRAPHY WITH IV CONTRAST,CT Dual Energy |
| CT COLON W | CT COLONOGRAPHY WITH IV CONTRAST |
| CT COLON W and WO | CT COLONOGRAPHY WO AND W IV CONT |
| CT COLON WO | CT COLONOGRAPHY WITHOUT IV CONTRAST |
| CT COLON WO | CT COLONOGRAPHY WITHOUT IV CONTRAST,CT COLONOGRAPHY WITHOUT IV CONTRAST |
| CT COLON WO | CT COLONOGRAPHY WO IV CONT |
| CT CORONARY FRACTIONAL FLOW RESERVE (FFR) | CT CORONARY FRACTIONAL FLOW RESERVE (FFR) |
| CT C-SPINE W | CT CERVICAL SPIN |
| CT C-SPINE W | CT CERVICAL SPINE W IV CONT |
| CT C-SPINE W | CT CERVICAL SPINE WITH IV CONTRAST |
| CT C-SPINE W | CT CERVICAL SPINE WITH IV CONTRAST,CT CERVICAL SPINE WITH IV CONTRAST |
| CT C-SPINE W | CT CERVICAL SPINE WITH IV CONTRAST,CT Dual Energy |
| CT C-SPINE W and WO | CT CERVICAL SPINE WITHOUT AND WITH IV CONTRAST |
| CT C-SPINE W and WO | CT CERVICAL SPINE WO AND W IV CONT |
| CT C-SPINE WO | CT CERVICAL SPINE WITHOUT IV CONTRAST |
| CT C-SPINE WO | CT CERVICAL SPINE WITHOUT IV CONTRAST,CT CERVICAL SPINE WITHOUT IV CONTRAST |
| CT C-SPINE WO | CT CERVICAL SPINE WITHOUT IV CONTRAST,CT CERVICAL SPINE WITHOUT IV CONTRAST,CT CERVICAL SPINE WITHOUT IV CONTRAST |
| CT C-SPINE WO | CT CERVICAL SPINE WITHOUT IV CONTRAST,CT CERVICAL SPINE WITHOUT IV CONTRAST,CT CERVICAL SPINE WITHOUT IV CONTRAST,CT CERVICAL SPINE WITHOUT IV CONTRAST |
| CT C-SPINE WO | CT CERVICAL SPINE WITHOUT IV CONTRAST,CT CERVICAL SPINE WITHOUT IV CONTRAST,CT Dual Energy |
| CT C-SPINE WO | CT CERVICAL SPINE WITHOUT IV CONTRAST,CT Dual Energy |
| CT C-SPINE WO | CT CERVICAL SPINE WITHOUT IV CONTRAST,MM Reading |
| CT C-SPINE WO | CT CERVICAL SPINE WO IV CONT |
| CT CYSTOGRAM WITH CONTRAST | CT CYSTOGRAM WITH CONTRAST |
| CT CYSTOGRAM WITH CONTRAST | CT CYSTOGRAM WITH CONTRAST,CT Dual Energy |
| CT DENTA SCAN | CT DENTA SCAN |
| CT Dual Energy | CT Dual Energy |
| CT ELBOW W | CT ELBOW LEFT WI |
| CT ELBOW W | CT ELBOW LEFT WITHOUT IV CONTRAST |
| CT ELBOW W | CT ELBOW RIGHT WITH IV CONTRAST |
| CT ELBOW W | CT ELBOW RIGHT WITH IV CONTRAST,CT Dual Energy |
| CT ELBOW WO | CT ELBOW LEFT WITH IV CONTRAST |
| CT ELBOW WO | CT ELBOW RIGHT WITHOUT IV CONTRAST |
| CT ELBOW WO | CT LT ELBOW WITHOUT IV CONTRAST |
| CT ENTEROCOLYSIS W | CT ENTEROCLYSIS W IV CONTRAST ONLY |
| CT ENTEROCOLYSIS W and WO | CT ENTEROCOLYSIS WO AND W IV CONT |
| CT ENTEROCOLYSIS WO | CT ENTEROCOLYSIS WITHOUT IV CONTRAST ONLY |
| CT ENTEROGRAPHY W | CT ENTEROGRAPHY WITH IV CONTRAST |
| CT ENTEROGRAPHY W | CT ENTEROGRAPHY WITH IV CONTRAST,CT Dual Energy |
| CT ENTEROGRAPHY W | CT ENTEROGRAPHY WITH IV CONTRAST,MM Reading |
| CT ENTEROGRAPHY WO | CT ENTEROGRAPHY WITHOUT IV CONTRAST |
| CT FEMUR W | CT FEMUR (THIGH) LEFT WITH IV CONTRAST |
| CT FEMUR W | CT FEMUR (THIGH) RIGHT WITH IV CONTRAST |
| CT FEMUR W | CT RT FEMUR (THIGH) WITH IV CONTRAST |
| CT FEMUR W and WO | CT FEMUR (THIGH) LEFT WITHOUT AND WITH IV CONTRAST |
| CT FEMUR WO | CT FEMUR (THIGH) LEFT WITHOUT IV CONTRAST |
| CT FEMUR WO | CT FEMUR (THIGH) LEFT WITHOUT IV CONTRAST,MM Reading |
| CT FEMUR WO | CT FEMUR (THIGH) RIGHT WITHOUT IV CONTRAST |
| CT FEMUR WO | CT FEMUR (THIGH) RIGHT WITHOUT IV CONTRAST,CT Dual Energy |
| CT FEMUR WO | CT LT FEMUR (THIGH) WITHOUT IV CONTRAST |
| CT FEMUR WO | CT RT FEMUR (THIGH) WITHOUT IV CONTRAST |
| CT FINE NEEDLE ASPIRATION BIOPSY EA ADD LESION | CT FINE NEEDLE ASPIRATION BIOPSY EA ADD LESION |
| CT FINE NEEDLE ASPIRATION BIOPSY FIRST LESION | CT FINE NEEDLE ASPIRATION BIOPSY FIRST LESION |
| CT FINGER WO | CT FINGER LEFT WITHOUT IV CONTRAST |
| CT FINGER WO | CT FINGER RIGHT WITHOUT IV CONTRAST |
| CT FOREARM W | CT FOREARM (RADIUS ULNA) LEFT WITH IV CONTRAST |
| CT FOREARM W | CT FOREARM (RADIUS ULNA) RIGHT WITH IV CONTRAST |
| CT FOREARM WO | CT FOREARM (RADIUS ULNA) LEFT WITHOUT IV CONTRAST |
| CT FOREARM WO | CT FOREARM (RADIUS ULNA) RIGHT WITHOUT IV CONTRAST |
| CT FOREFOOT W | CT FOREFOOT LEFT WITH IV CONTRAST |
| CT FOREFOOT W | CT FOREFOOT LEFT WITH IV CONTRAST,CT FOREFOOT LEFT WITH IV CONTRAST |
| CT FOREFOOT W | CT FOREFOOT RIGHT WITH IV CONTRAST |
| CT FOREFOOT W | CT FOREFOOT RIGHT WITH IV CONTRAST,CT FOREFOOT RIGHT WITH IV CONTRAST,CT FOREFOOT RIGHT WITH IV CONTRAST,CT FOREFOOT RIGHT WITH IV CONTRAST,CT FOREFOOT RIGHT WITH IV CONTRAST |
| CT FOREFOOT W and WO | CT FOREFOOT RIGHT WITHOUT AND WITH IV CONTRAST |
| CT FOREFOOT WO | CT FOREFOOT LEFT WITHOUT IV CONTRAST |
| CT FOREFOOT WO | CT FOREFOOT LEFT WITHOUT IV CONTRAST,CT Dual Energy |
| CT FOREFOOT WO | CT FOREFOOT RIGHT WITHOUT IV CONTRAST |
| CT FOREFOOT WO | CT FOREFOOT RIGHT WITHOUT IV CONTRAST,CT Dual Energy |
| CT FOREFOOT WO | CT LT FOREFOOT WITHOUT IV CONTRAST |
| CT GUIDANCE FOR BIOPSY | CT GUIDANCE FOR BIOPSY |
| CT GUIDANCE FOR IR PROCEDURE | CT GUIDANCE FOR IR PROCEDURE |
| CT GUIDE TISSUE ABLATION | CT GUIDE TISSUE ABLATION |
| CT GUIDE/MONITOR PARENCHYMAL TISSUE ABLATION | CT GUIDE/MONITOR PARENCHYMAL TISSUE ABLATION |
| CT GUIDED ABSCESS DRAIN CORE NEEDLE | CT GUIDED ABSCESS DRAIN CORE NEEDLE |
| CT GUIDED ABSCESS DRAIN CORE NEEDLE | CT GUIDED ABSCESS DRAIN CORE NEEDLE,CT GUIDED ABSCESS DRAIN CORE NEEDLE |
| CT GUIDED BONE BIOPSY DEEP CORE NEEDLE | CT GUIDED BONE BIOPSY DEEP CORE NEEDLE |
| CT GUIDED BONE MARROW BIOPSY | CT GUIDED BONE MARROW BIOPSY |
| CT GUIDED BONE MARROW BIOPSY | CT GUIDED BONE MARROW BIOPSY,CT Dual Energy |
| CT GUIDED CHEST/PLEURA BIOPSY CORE NEEDLE | CT GUIDED CHEST/PLEURA BIOPSY CORE NEEDLE |
| CT GUIDED LIVER BIOPSY CORE NEEDLE | CT GUIDED LIVER BIOPSY CORE NEEDLE |
| CT GUIDED LUNG BIOPSY CORE NEEDLE | CT GUIDED LUNG BIOPSY CORE NEEDLE |
| CT GUIDED LUNG BIOPSY CORE NEEDLE | CT GUIDED LUNG BIOPSY CORE NEEDLE,CT Dual Energy |
| CT GUIDED NEEDLE PLACEMENT | CT GUIDED NEEDLE PLACEMENT |
| CT GUIDED RENAL BIOPSY CORE NEEDLE | CT GUIDED RENAL BIOPSY CORE NEEDLE |
| CT GUIDED RETROPERITONEAL BIOPSY CORE NEEDLE | CT GUIDED RETROPERITONEAL BIOPSY CORE NEEDLE |
| CT HAND W | CT HAND LEFT WITH IV CONTRAST |
| CT HAND W | CT HAND LEFT WITH IV CONTRAST,CT HAND LEFT WITH IV CONTRAST |
| CT HAND W | CT HAND RIGHT WITH IV CONTRAST |
| CT HAND WO | CT HAND LEFT WITHOUT IV CONTRAST |
| CT HAND WO | CT HAND LEFT WITHOUT IV CONTRAST,CT Dual Energy |
| CT HAND WO | CT HAND RIGHT WITHOUT IV CONTRAST |
| CT HAND WO | CT HAND RIGHT WITHOUT IV CONTRAST,CT Dual Energy |
| CT HEAD WO | CT 3D HEAD WITHOUT IV CONTRAST |
| CT HEAD WO | CT 3D HEAD WITHOUT IV CONTRAST,CT 3D HEAD WITHOUT IV CONTRAST |
| CT HEAD WO | CT 3D HEAD WITHOUT IV CONTRAST,CT Dual Energy |
| CT HEAD WO | CT 3D HEAD WITHOUT IV CONTRAST,MM Reading |
| CT HEART CORONARY ARTERIES AND BYPASS GRAFTS W | CT HEART CORONAR |
| CT HEART CORONARY ARTERIES AND BYPASS GRAFTS W | CT HEART CORONARY ARTERIES & BYPASS GRAFTS WITH IV CONTRAST |
| CT HEART CORONARY ARTERIES AND BYPASS GRAFTS W | CT HEART CORONARY ARTERIES & BYPASS GRAFTS WITH IV CONTRAST,CT Cardiac |
| CT HEART CORONARY ARTERIES AND BYPASS GRAFTS W | CT HEART CORONARY ARTERIES & BYPASS GRAFTS WITH IV CONTRAST,CT CaScoring |
| CT HEART CORONARY ARTERIES AND BYPASS GRAFTS W | CT HEART CORONARY ARTERIES & BYPASS GRAFTS WITH IV CONTRAST,CT Dual Energy |
| CT HEART CORONARY ARTERIES AND BYPASS GRAFTS W | CT HEART CORONARY ARTERIES & BYPASS GRAFTS WITH IV CONTRAST,CT HEART CORONARY ARTERIES & BYPASS GRAFTS WITH IV CONTRAST |
| CT HEART CORONARY ARTERIES AND BYPASS GRAFTS W | CT HEART CORONARY ARTERIES AN BYPASS GRAFTS W IV CONTRAST |
| CT HEART FOR CORONARY CALCIUM - RESEARCH ONLY | CT HEART FOR CORONARY CALCIUM - REAEARCH ONLY |
| CT HEART FOR CORONARY CALCIUM - RESEARCH ONLY | CT HEART FOR CORONARY CALCIUM - RESEARCH ONLY |
| CT HEART FOR QUANTIFICATION OF CORONARY CALCIUM | CT HEART FOR QUA |
| CT HEART FOR QUANTIFICATION OF CORONARY CALCIUM | CT HEART FOR QUANTIFICATION OF CORONARY CALCIUM |
| CT HEART FOR QUANTIFICATION OF CORONARY CALCIUM | CT HEART FOR QUANTIFICATION OF CORONARY CALCIUM,CT CaScoring |
| CT HEART FOR QUANTIFICATION OF CORONARY CALCIUM | CT HEART FOR QUANTIFICATION OF CORONARY CALCIUM,CT HEART FOR QUANTIFICATION OF CORONARY CALCIUM |
| CT HEART FOR STRUCTURE AND MORPHOLOGY WITH IV CONTRAST | CT HEART FOR STRUCTURE AND MORPHOLOGY WITH IV CONTRAST |
| CT HEART FOR STRUCTURE AND MORPHOLOGY WITH IV CONTRAST | CT HEART FOR STRUCTURE AND MORPHOLOGY WITH IV CONTRAST,CT Cardiac |
| CT HEART FOR STRUCTURE AND MORPHOLOGY WITH IV CONTRAST | CT HEART FOR STRUCTURE AND MORPHOLOGY WITH IV CONTRAST,CT HEART FOR STRUCTURE AND MORPHOLOGY WITH IV CONTRAST |
| CT HEART FOR STRUCTURE AND MORPHOLOGY WITH IV CONTRAST | CT HEART STRUCTURE & MORPHOLOGY WITH IV CONTRAST - CHD,CT Cardiac |
| CT HEART FOR STRUCTURE AND MORPHOLOGY WITH IV CONTRAST | CT HEART STRUCTURE & MORPHOLOGY WITH IV CONTRAST - CHD,CT Dual Energy |
| CT HEART FOR STRUCTURE AND MORPHOLOGY WITH IV CONTRAST | CT HEART STRUCTURE & MORPHOLOGY WITH IV CONTRAST - CHD,CT HEART STRUCTURE & MORPHOLOGY WITH IV CONTRAST - CHD |
| CT HEART FOR STRUCTURE AND MORPHOLOGY WITH IV CONTRAST | CT HEART STRUCTURE & MORPHOLOGY WITH IV CONTRAST - CHD,CT HEART STRUCTURE & MORPHOLOGY WITH IV CONTRAST - CHD,CT HEART STRUCTURE & MORPHOLOGY WITH IV CONTRAST - CHD |
| CT HEART SCORE - PROMOTION | CT HEART SCORE - PROMOTION |
| CT HEART SCORE - PROMOTION | CT HEART SCORE - PROMOTION,CT CaScoring |
| CT HEART SCORE WITHOUT IV CONTRAST- PROMOTION | CT HEART SCORE WITHOUT IV CONTRAST- PROMOTION |
| CT HEART SCORE WITHOUT IV CONTRAST- PROMOTION | CT HEART SCORE WITHOUT IV CONTRAST- PROMOTION,CT CaScoring |
| CT HEART STRUCTURE & MORPHOLOGY WITH IV CONTRAST - CHD | CT HEART STRUCTU |
| CT HEART STRUCTURE & MORPHOLOGY WITH IV CONTRAST - CHD | CT HEART STRUCTURE & MORPHOLOGY WITH IV CONTRAST - CHD |
| CT HEART STRUCTURE AND MORPHOLOGY WITH IV CONTRAST - CHD | CT HEART STRUCTURE AND MORPHOLOGY WITH IV CONTRAST - CHD |
| CT HEART WO | CT HEART WITHOUT IV CONTRAST FOR CORONARY CALCIUM - RESEARCH O*,CT CaScoring |
| CT HEART WO | CT HEART WO IV CONT |
| CT HEART WO COR CAL QUANT | CT HEART WITHOUT IV CONTRAST FOR QUANTIFICATION OF CORONARY CAL* |
| CT HEART WO COR CAL QUANT | CT HEART WITHOUT IV CONTRAST FOR QUANTIFICATION OF CORONARY CAL*,CT CaScoring |
| CT HIP W | CT HIP LEFT WITH IV CONTRAST |
| CT HIP W | CT HIP RIGHT WIT |
| CT HIP W | CT HIP RIGHT WITH IV CONTRAST |
| CT HIP W and WO | CT HIP LEFT WITHOUT AND WITH IV CONTRAST |
| CT HIP W and WO | CT HIP RIGHT WITHOUT AND WITH IV CONTRAST |
| CT HIP WO | CT HIP LEFT WITHOUT IV CONTRAST |
| CT HIP WO | CT HIP LEFT WITHOUT IV CONTRAST,CT Dual Energy |
| CT HIP WO | CT HIP LEFT WITHOUT IV CONTRAST,CT HIP LEFT WITHOUT IV CONTRAST |
| CT HIP WO | CT HIP RIGHT WITHOUT IV CONTRAST |
| CT HIP WO | CT HIP RIGHT WITHOUT IV CONTRAST,CT Dual Energy |
| CT HIP WO | CT HIP RIGHT WITHOUT IV CONTRAST,CT HIP RIGHT WITHOUT IV CONTRAST |
| CT HIP WO | CT LT HIP WITHOUT IV CONTRAST |
| CT HIP WO | CT RT HIP WITHOUT IV CONTRAST |
| CT HUMERUS W | CT HUMERUS LEFT WITH IV CONTRAST |
| CT HUMERUS W | CT HUMERUS RIGHT WITH IV CONTRAST |
| CT HUMERUS W and WO | CT HUMERUS RIGHT WITHOUT AND WITH IV CONTRAST |
| CT HUMERUS WO | CT HUMERUS LEFT WITHOUT IV CONTRAST |
| CT HUMERUS WO | CT HUMERUS LEFT WITHOUT IV CONTRAST,CT HUMERUS LEFT WITHOUT IV CONTRAST |
| CT HUMERUS WO | CT HUMERUS RIGHT WITHOUT IV CONTRAST |
| CT KIDNEY STONE PROTOCOL | CT KIDNEY STONE PROTOCOL |
| CT KNEE W | CT KNEE LEFT WIT |
| CT KNEE W | CT KNEE LEFT WITH IV CONTRAST |
| CT KNEE W | CT KNEE LEFT WITH IV CONTRAST,CT Dual Energy |
| CT KNEE W | CT KNEE RIGHT WITH IV CONTRAST |
| CT KNEE W | CT KNEE RIGHT WITH IV CONTRAST,CT KNEE RIGHT WITH IV CONTRAST |
| CT KNEE W and WO | CT KNEE LEFT WITHOUT AND WITH IV CONTRAST |
| CT KNEE W and WO | CT KNEE RIGHT WITHOUT AND WITH IV CONTRAST |
| CT KNEE WO | CT KNEE LEFT WITHOUT IV CONTRAST |
| CT KNEE WO | CT KNEE LEFT WITHOUT IV CONTRAST,CT Dual Energy |
| CT KNEE WO | CT KNEE LEFT WITHOUT IV CONTRAST,CT KNEE LEFT WITHOUT IV CONTRAST,CT KNEE LEFT WITHOUT IV CONTRAST |
| CT KNEE WO | CT KNEE RIGHT WITHOUT IV CONTRAST |
| CT KNEE WO | CT KNEE RIGHT WITHOUT IV CONTRAST,CT Dual Energy |
| CT KNEE WO | CT KNEE RIGHT WITHOUT IV CONTRAST,CT KNEE RIGHT WITHOUT IV CONTRAST |
| CT KNEE WO | CT LT KNEE WITHOUT IV CONTRAST |
| CT KNEE WO | CT RT KNEE WITHOUT IV CONTRAST |
| CT LEG W | CT LEG (TIBIA/FI |
| CT LEG W | CT LEG (TIBIA/FIBULA) LEFT WITH IV CONTRAST |
| CT LEG W | CT LEG (TIBIA/FIBULA) LEFT WITH IV CONTRAST,CT Dual Energy |
| CT LEG W | CT LEG (TIBIA/FIBULA) RIGHT WITH IV CONTRAST |
| CT LEG W | CT LEG (TIBIA/FIBULA) RIGHT WITH IV CONTRAST,CT Dual Energy |
| CT LEG W | CT LEG (TIBIA/FIBULA) RIGHT WITH IV CONTRAST,CT LEG (TIBIA/FIBULA) RIGHT WITH IV CONTRAST |
| CT LEG W | CT LEG (TIBIA-FIBULA) RIGHT WITH IV CONTRAST |
| CT LEG W | CT LT LEG (TIBIA-FIBULA) WITH IV CONTRAST |
| CT LEG W and WO | CT LEG (TIBIA-FIBULA) LEFT WITHOUT AND WITH IV CONTRAST |
| CT LEG W and WO | CT LEG (TIBIA-FIBULA) RIGHT WITHOUT AND WITH IV CONTRAST |
| CT LEG WO | CT LEG (TIBIA/FIBULA) LEFT WITHOUT IV CONTRAST |
| CT LEG WO | CT LEG (TIBIA/FIBULA) LEFT WITHOUT IV CONTRAST,CT Dual Energy |
| CT LEG WO | CT LEG (TIBIA/FIBULA) LEFT WITHOUT IV CONTRAST,CT Dual Energy,CT LEG (TIBIA/FIBULA) LEFT WITHOUT IV CONTRAST |
| CT LEG WO | CT LEG (TIBIA/FIBULA) RIGHT WITHOUT IV CONTRAST |
| CT LEG WO | CT LEG (TIBIA/FIBULA) RIGHT WITHOUT IV CONTRAST,CT Dual Energy |
| CT LEG WO | CT LEG (TIBIA-FIBULA) LEFT WITHOUT IV CONTRAST |
| CT LEG WO | CT LEG (TIBIA-FIBULA) RIGHT WITHOUT IV CONTRAST |
| CT LEG WO | CT LT LEG (TIBIA-FIBULA) WITHOUT IV CONTRAST |
| CT LEG WO | CT RT LEG (TIBIA-FIBULA) WITHOUT IV CONTRAST |
| CT LOWER EX W | CT LEFT LOWER EX |
| CT LOWER EX W | CT LEFT LOWER EXTREMITY W IV CONT |
| CT LOWER EX W | CT LEFT UPPER EXTREMITY W IV CONT |
| CT LOWER EX W | CT RIGHT LOWER EXTREMITY W IV CONT |
| CT LOWER EX WO | CT LEFT LOWER EXTREMITY WO IV CONT |
| CT LOWER EX WO | CT LEFT UPPER EXTREMITY WO IV CONT |
| CT LOWER EX WO | CT RIGHT LOWER EXTREMITY WO IV CONT |
| CT L-SPINE W | CT LUMBAR SPINE |
| CT L-SPINE W | CT LUMBAR SPINE W IV CONT |
| CT L-SPINE W | CT LUMBAR SPINE WITH IV CONTRAST |
| CT L-SPINE W | CT LUMBAR SPINE WITH IV CONTRAST,CT Dual Energy |
| CT L-SPINE W | CT LUMBAR SPINE WITH IV CONTRAST,CT LUMBAR SPINE WITH IV CONTRAST |
| CT L-SPINE W | CT LUMBAR SPINE WITH IV CONTRAST,MM Reading |
| CT L-SPINE W and WO | CT LUMBAR SPINE WITHOUT AND WITH IV CONTRAST |
| CT L-SPINE W and WO | CT LUMBAR SPINE WO AND W IV CONT |
| CT L-SPINE WO | CT LUMBAR SPINE WITHOUT IV CONTRAST |
| CT L-SPINE WO | CT LUMBAR SPINE WITHOUT IV CONTRAST,CT Dual Energy |
| CT L-SPINE WO | CT LUMBAR SPINE WITHOUT IV CONTRAST,CT LUMBAR SPINE WITHOUT IV CONTRAST |
| CT L-SPINE WO | CT LUMBAR SPINE WITHOUT IV CONTRAST,CT LUMBAR SPINE WITHOUT IV CONTRAST,CT Dual Energy |
| CT L-SPINE WO | CT LUMBAR SPINE WITHOUT IV CONTRAST,MM Reading |
| CT L-SPINE WO | CT LUMBAR SPINE WO IV CONT |
| CT MASTOIDS W | CT MASTOIDS WITH IV CONTRAST |
| CT MASTOIDS WO | CT MASTOIDS WITHOUT IV CONTRAST |
| CT MAXILLO W | CT MAXILLOFACIAL |
| CT MAXILLO W | CT MAXILLOFACIAL W IV CONT |
| CT MAXILLO W | CT MAXILLOFACIAL WITH IV CONTRAST |
| CT MAXILLO W | CT MAXILLOFACIAL WITH IV CONTRAST,CT Dual Energy |
| CT MAXILLO W | CT MAXILLOFACIAL WITH IV CONTRAST,CT MAXILLOFACIAL WITH IV CONTRAST |
| CT MAXILLO W | CT MAXILLOFACIAL WITH IV CONTRAST,CT MAXILLOFACIAL WITH IV CONTRAST,CT Dual Energy |
| CT MAXILLO W | CT MAXILLOFACIAL WITH IV CONTRAST,MM Reading |
| CT MAXILLO W | CT MAXILLOFACIAL,CT MAXILLOFACIAL |
| CT MAXILLO W and WO | CT MAXILLOFACIAL WITHOUT AND WITH IV CONTRAST |
| CT MAXILLO W and WO | CT MAXILLOFACIAL WO AND W IV CONT |
| CT MAXILLO WO | CT MAXILLOFACIAL WITHOUT IV CONTRAST |
| CT MAXILLO WO | CT MAXILLOFACIAL WO IV CONT |
| CT MAXILLO-SINUS W | CT MAXILLOFACIAL-SINUS WITH IV CONTRAST |
| CT MAXILLO-SINUS W | CT MAXILLOFACIAL-SINUS WITH IV CONTRAST,CT MAXILLOFACIAL-SINUS WITH IV CONTRAST |
| CT MAXILLO-SINUS W and WO | CT MAXILLOFACIAL-SINUS WITHOUT AND WITH IV CONTRAST |
| CT MAXILLO-SINUS WO | CT MAXILLOFACIAL-SINUS WITHOUT IV CONTRAST |
| CT MAXILLO-SINUS WO | CT MAXILLOFACIAL-SINUS WITHOUT IV CONTRAST,CT Dual Energy |
| CT MAXILLO-SINUS WO | CT MAXILLOFACIAL-SINUS WITHOUT IV CONTRAST,CT MAXILLOFACIAL-SINUS WITHOUT IV CONTRAST |
| CT MAXILLO-SINUS WO | CT MAXILLOFACIAL-SINUS WITHOUT IV CONTRAST,CT MAXILLOFACIAL-SINUS WITHOUT IV CONTRAST,CT Dual Energy |
| CT MAXILLO-SINUS WO | CT MAXILLOFACIAL-SINUS WITHOUT IV CONTRAST,MM Reading |
| CT NECK W | CT SOFT TISSUE N |
| CT NECK W | CT SOFT TISSUE NECK WITH IV CONTRAST |
| CT NECK W | CT SOFT TISSUE NECK WITH IV CONTRAST,CT Dual Energy |
| CT NECK W | CT SOFT TISSUE NECK WITH IV CONTRAST,CT SOFT TISSUE NECK WITH IV CONTRAST |
| CT NECK W | CT SOFT TISSUE NECK WITH IV CONTRAST,CT SOFT TISSUE NECK WITH IV CONTRAST,CT Dual Energy |
| CT NECK W | CT SOFT TISSUE NECK WITH IV CONTRAST,CT SOFT TISSUE NECK WITH IV CONTRAST,CT SOFT TISSUE NECK WITH IV CONTRAST |
| CT NECK W | CT SOFT TISSUE NECK WITH IV CONTRAST,MM Reading |
| CT NECK W and WO | CT SOFT TISSUE NECK WITHOUT AND WITH IV CONTRAST |
| CT NECK W and WO | CT SOFT TISSUE NECK WITHOUT AND WITH IV CONTRAST,CT Dual Energy |
| CT NECK W and WO | CT SOFT TISSUE NECK WO AND W IV CONT |
| CT NECK WO | CT SOFT TISSUE NECK WITHOUT IV CONTRAST |
| CT NECK WO | CT SOFT TISSUE NECK WITHOUT IV CONTRAST,CT Dual Energy |
| CT NECK WO | CT SOFT TISSUE NECK WITHOUT IV CONTRAST,CT SOFT TISSUE NECK WITHOUT IV CONTRAST |
| CT NECK WO | CT SOFT TISSUE NECK WO IV CONT |
| CT ORBITS W | CT ORBITS W IV CONT |
| CT ORBITS W | CT ORBITS WITH IV CONTRAST |
| CT ORBITS W | CT ORBITS WITH IV CONTRAST,CT Dual Energy |
| CT ORBITS W | CT ORBITS WITH IV CONTRAST,CT ORBITS WITH IV CONTRAST |
| CT ORBITS W and WO | CT ORBITS WITHOUT AND WITH IV CONTRAST |
| CT ORBITS W and WO | CT ORBITS WO AND W IV CONT |
| CT ORBITS WO | CT ORBITS WITHOUT IV CONTRAST |
| CT ORBITS WO | CT ORBITS WITHOUT IV CONTRAST,CT Dual Energy |
| CT ORBITS WO | CT ORBITS WO IV CONT |
| CT PELVIS W | CT PELVIS W IV CONT |
| CT PELVIS W | CT PELVIS WITH IV CONTRAST |
| CT PELVIS W | CT PELVIS WITH IV CONTRAST,CT Dual Energy |
| CT PELVIS W | CT PELVIS WITH IV CONTRAST,CT PELVIS WITH IV CONTRAST |
| CT PELVIS W | CT PELVIS WITH IV CONTRAST,MM Reading |
| CT PELVIS W and WO | CT PELVIS WITHOUT AND WITH IV CONTRAST |
| CT PELVIS W and WO | CT PELVIS WO AND W IV CONT |
| CT PELVIS WO | CT PELVIS WITHOUT IV CONTRAST |
| CT PELVIS WO | CT PELVIS WITHOUT IV CONTRAST,CT Dual Energy |
| CT PELVIS WO | CT PELVIS WITHOUT IV CONTRAST,CT PELVIS WITHOUT IV CONTRAST |
| CT PELVIS WO | CT PELVIS WO IV CONT |
| CT PERCUTANEOUS DRAINAGE | CT PERCUTANEOUS DRAINAGE |
| CT SACROILIAC W | CT SACROILIAC JOINT ( BILATERAL) WITH IV CONTRAST |
| CT SACROILIAC WO | CT SACROILIAC JOINT ( BILATERAL) WITHOUT IV CONTRAST |
| CT SACROILIAC WO | CT SACROILIAC JOINT (BILATERAL) WITHOUT IV CONTRAST |
| CT SCAPULA W | CT SCAPULA LEFT WITH IV CONTRAST |
| CT SCAPULA W | CT SCAPULA RIGHT WITH IV CONTRAST |
| CT SCAPULA WO | CT SCAPULA LEFT WITHOUT IV CONTRAST |
| CT SCAPULA WO | CT SCAPULA RIGHT WITHOUT IV CONTRAST |
| CT SHOULDER W | CT SHOULDER LEFT WITH IV CONTRAST |
| CT SHOULDER W | CT SHOULDER RIGHT WITH IV CONTRAST |
| CT SHOULDER W | CT SHOULDER RIGHT WITH IV CONTRAST,CT SHOULDER RIGHT WITH IV CONTRAST |
| CT SHOULDER W and WO | CT SHOULDER LEFT WITHOUT AND WITH IV CONTRAST |
| CT SHOULDER W and WO | CT SHOULDER RIGHT WITHOUT AND WITH IV CONTRAST |
| CT SHOULDER WO | CT LT SHOULDER WITHOUT IV CONTRAST |
| CT SHOULDER WO | CT RIGHT SHOULDER WITHOUT IV CONTRAST |
| CT SHOULDER WO | CT RT SHOULDER WITHOUT IV CONTRAST |
| CT SHOULDER WO | CT SHOULDER LEFT WITHOUT IV CONTRAST |
| CT SHOULDER WO | CT SHOULDER LEFT WITHOUT IV CONTRAST,MM Reading |
| CT SHOULDER WO | CT SHOULDER RIGHT WITHOUT IV CONTRAST |
| CT SHOULDER WO | CT SHOULDER RIGHT WITHOUT IV CONTRAST,CT Dual Energy |
| CT SHOULDER WO | CT SHOULDER RIGHT WITHOUT IV CONTRAST,CT SHOULDER RIGHT WITHOUT IV CONTRAST,CT Dual Energy |
| CT SINUS W | CT SINUS W IV CONT |
| CT SINUS W and WO | CT SINUS WO AND W IV CONT |
| CT SINUS WO | CT SINUS WITHOUT |
| CT SINUS WO | CT SINUS WO IV CONT |
| CT SINUS WO | CT SINUSES LIMITED WITHOUT IV CONTRAST |
| CT SLEEP | CT SLEEP PROTOCOL |
| CT SLEEP | CT SOFT TISSUE NECK - SLEEP PROTOCOL |
| CT SLEEP | CT SOFT TISSUE NECK WITHOUT IV CONTRAST - SLEEP PROTOCOL |
| CT STEREOTATIC GUIDIANCE BIOPSY | CT STEREOTATIC GUIDIANCE BIOPSY |
| CT STEREOTATIC GUIDIANCE BIOPSY | CT STEREOTATIC GUIDIANCE BIOPSY |
| CT STERNUM W | CT STERNUM WITH IV CONTRAST |
| CT STERNUM W and WO | CT STERNUM WITHOUT AND WITH IV CONTRAST |
| CT STERNUM WO | CT STERNUM WITHOUT IV CONTRAST |
| CT TEMPORAL | CT TEMPORAL BONE |
| CT TEMPORAL W | CT TEMPORAL BONES W IV CONT |
| CT TEMPORAL W | CT TEMPORAL BONES WITH IV CONTRAST |
| CT TEMPORAL W | CT TEMPORAL BONES WITH IV CONTRAST,CT TEMPORAL BONES WITH IV CONTRAST |
| CT TEMPORAL W | CT TEMPORAL BONES WITH IV CONTRAST,CT TEMPORAL BONES WITH IV CONTRAST,CT TEMPORAL BONES WITH IV CONTRAST |
| CT TEMPORAL W and WO | CT TEMPORAL BONES WITHOUT AND WITH IV CONTRAST |
| CT TEMPORAL W and WO | CT TEMPORAL BONES WO AND W IV CONT |
| CT TEMPORAL WO | CT TEMPORAL BONES WITHOUT IV CONTRAST |
| CT TEMPORAL WO | CT TEMPORAL BONES WITHOUT IV CONTRAST,CT Dual Energy |
| CT TEMPORAL WO | CT TEMPORAL BONES WO IV CONT |
| CT THUMB WO | CT THUMB LEFT WITHOUT IV CONTRAST |
| CT T-SPINE W | CT THORACIC SPIN |
| CT T-SPINE W | CT THORACIC SPINE W IV CONT |
| CT T-SPINE W | CT THORACIC SPINE WITH IV CONTRAST |
| CT T-SPINE W | CT THORACIC SPINE WITH IV CONTRAST,CT Dual Energy |
| CT T-SPINE W | CT THORACIC SPINE WITH IV CONTRAST,CT THORACIC SPINE WITH IV CONTRAST |
| CT T-SPINE W and WO | CT THORACIC SPINE WO AND W IV CONT |
| CT T-SPINE WO | CT THORACIC SPINE WITHOUT IV CONTRAST |
| CT T-SPINE WO | CT THORACIC SPINE WITHOUT IV CONTRAST,CT Dual Energy |
| CT T-SPINE WO | CT THORACIC SPINE WITHOUT IV CONTRAST,CT THORACIC SPINE WITHOUT IV CONTRAST |
| CT T-SPINE WO | CT THORACIC SPINE WITHOUT IV CONTRAST,CT THORACIC SPINE WITHOUT IV CONTRAST,CT Dual Energy |
| CT T-SPINE WO | CT THORACIC SPINE WITHOUT IV CONTRAST,CT THORACIC SPINE WITHOUT IV CONTRAST,MM Reading |
| CT T-SPINE WO | CT THORACIC SPINE WITHOUT IV CONTRAST,MM Reading |
| CT T-SPINE WO | CT THORACIC SPINE WO IV CONT |
| CT UPPER EX W | CT RIGHT UPPER EXTREMITY W IV CONT |
| CT UPPER EX WO | CT RIGHT UPPER EXTREMITY WO IV CONT |
| CT UROGRAM W | CT UROGRAM W IV CONT |
| CT UROGRAM W | CT UROGRAM WITH IV CONTRAST |
| CT UROGRAM W | CT UROGRAM WITH IV CONTRAST,MM Reading |
| CT UROGRAM W and WO | CT UROGRAM WITHOUT AND WITH IV CONTRAST |
| CT UROGRAM W and WO | CT UROGRAM WITHOUT AND WITH IV CONTRAST,CT Dual Energy |
| CT UROGRAM W and WO | CT UROGRAM WITHOUT AND WITH IV CONTRAST,CT UROGRAM WITHOUT AND WITH IV CONTRAST |
| CT UROGRAM W and WO | CT UROGRAM WITHOUT AND WITH IV CONTRAST,MM Reading |
| CT VENOGRAM HEAD | CT VENOGRAM HEAD |
| CT VENOGRAM HEAD | CT VENOGRAM HEAD,CT VENOGRAM HEAD |
| CT VENOGRAM- HEAD WITH AND WITHOUT IV CONTRAST | CT VENOGRAM- HEAD WITH AND WITHOUT IV CONTRAST |
| CT VENOGRAM- HEAD WITH AND WITHOUT IV CONTRAST | CT VENOGRAM- HEAD WITH AND WITHOUT IV CONTRAST,CT Dual Energy |
| CT VISUAL TRACKING WO | CT VISUAL TRACKI |
| CT VISUAL TRACKING WO | CT VISUAL TRACKING |
| CT VISUAL TRACKING WO | CT VISUAL TRACKING WITHOUT IV CONTRAST |
| CT VISUAL TRACKING WO | CT VISUAL TRACKING WITHOUT IV CONTRAST,CT Dual Energy |
| CT VISUAL TRACKING WO | CT VISUAL TRACKING WITHOUT IV CONTRAST,CT VISUAL TRACKING WITHOUT IV CONTRAST |
| CT VISUAL TRACKING WO | CT VISUAL TRACKING WITHOUT IV CONTRAST,CT VISUAL TRACKING WITHOUT IV CONTRAST,CT Dual Energy |
| CT VISUAL TRACKING WO | CT VISUAL TRACKING WITHOUT IV CONTRAST,MM Reading |
| CT VISUAL TRACKING WO | CT VISUAL TRACKING,CT VISUAL TRACKING |
| CT WRIST W | CT LT WRIST WITH IV CONTRAST |
| CT WRIST W | CT WRIST RIGHT WITH IV CONTRAST |
| CT WRIST W and WO | CT WRIST LEFT WITHOUT AND WITH IV CONTRAST |
| CT WRIST WO | CT RT WRIST WITHOUT IV CONTRAST |
| CT WRIST WO | CT WRIST LEFT WITHOUT IV CONTRAST |
| CT WRIST WO | CT WRIST RIGHT WITHOUT IV CONTRAST |
| CT WRIST WO | CT WRIST RIGHT WITHOUT IV CONTRAST,CT Dual Energy |
| CTA ABD | CT ANGIOGRAPHY A |
| CTA ABD W | CT ANGIOGRAPHY ABDOMEN |
| CTA ABD W | CT ANGIOGRAPHY ABDOMEN WITH IV CONTRAST |
| CTA ABD W | CT ANGIOGRAPHY ABDOMEN WITH IV CONTRAST,CT Dual Energy |
| CTA ABD W | CT ANGIOGRAPHY ABDOMEN WITH IV CONTRAST,MM Reading |
| CTA ABD W | CT ANGIOGRAPHY ABDOMEN,CT ANGIOGRAPHY ABDOMEN,CT ANGIOGRAPHY ABDOMEN,CT ANGIOGRAPHY ABDOMEN |
| CTA ABD/PELV W | CT ANGIOGRAPHY ABD/PELVIS WITH IV CONTRAST |
| CTA ABD/PELV W | CT ANGIOGRAPHY ABD/PELVIS WITH IV CONTRAST,CT ANGIOGRAPHY ABD/PELVIS WITH IV CONTRAST |
| CTA ABD/PELV W | CT ANGIOGRAPHY ABD/PELVIS WITH IV CONTRAST,CT ANGIOGRAPHY ABD/PELVIS WITH IV CONTRAST,CT ANGIOGRAPHY ABD/PELVIS WITH IV CONTRAST,CT ANGIOGRAPHY ABD/PELVIS WITH IV CONTRAST |
| CTA ABD/PELV W | CT ANGIOGRAPHY ABD/PELVIS WITH IV CONTRAST,CT ANGIOGRAPHY ABD/PELVIS WITH IV CONTRAST,CT Dual Energy |
| CTA ABD/PELV W | CT ANGIOGRAPHY ABD/PELVIS WITH IV CONTRAST,CT Dual Energy |
| CTA ABD/PELV W | CT ANGIOGRAPHY ABD/PELVIS WITH IV CONTRAST,CT Dual Energy,CT ANGIOGRAPHY ABD/PELVIS WITH IV CONTRAST |
| CTA ABD/PELV W | CT ANGIOGRAPHY ABD/PELVIS WITH IV CONTRAST,MM Reading |
| CTA ABD/PELV W | CT ANGIOGRAPHY ABD/PELVIS WITH IV CONTRAST,MM Reading |
| CTA ABD/PELV W | CT ANGIOGRAPHY ABDOMEN AND PELVIS |
| CTA ABD/PELV W | CT ANGIOGRAPHY ABDOMEN AND PELVIS,CT ANGIOGRAPHY ABDOMEN AND PELVIS,CT ANGIOGRAPHY ABDOMEN AND PELVIS |
| CTA ABD/PELV W and WO | CT ANGIOGRAPHY ABD PELVIS WITHOUT WITH IV CONTRAST |
| CTA ABD/PELV W and WO | CT ANGIOGRAPHY ABD/PELVIS WITHOUT & WITH IV CONTRAST |
| CTA ABD/PELV W and WO | CT ANGIOGRAPHY ABD/PELVIS WITHOUT & WITH IV CONTRAST,CT ANGIOGRAPHY ABD/PELVIS WITHOUT & WITH IV CONTRAST |
| CTA ABD/PELV W and WO | CT ANGIOGRAPHY ABD/PELVIS WITHOUT & WITH IV CONTRAST,CT ANGIOGRAPHY ABD/PELVIS WITHOUT & WITH IV CONTRAST,CT ANGIOGRAPHY ABD/PELVIS WITHOUT & WITH IV CONTRAST |
| CTA ABD/PELV W and WO | CT ANGIOGRAPHY ABD/PELVIS WITHOUT & WITH IV CONTRAST,CT ANGIOGRAPHY ABD/PELVIS WITHOUT & WITH IV CONTRAST,CT ANGIOGRAPHY ABD/PELVIS WITHOUT & WITH IV CONTRAST,CT ANGIOGRAPHY ABD/PELVIS WITHOUT & WITH IV CONTRAST |
| CTA ABD/PELV W and WO | CT ANGIOGRAPHY ABD/PELVIS WITHOUT & WITH IV CONTRAST,MM Reading |
| CTA AORTO-ILEOFEMORAL RUNOFF W | CT ANGIOGRAPHY AORTO-ILEOFEMORAL RUNOFF |
| CTA AORTO-ILEOFEMORAL RUNOFF W | CT ANGIOGRAPHY AORTO-ILEOFEMORAL RUNOFF WITH IV CONTRAST |
| CTA AORTO-ILEOFEMORAL RUNOFF W | CT ANGIOGRAPHY AORTO-ILEOFEMORAL RUNOFF WITH IV CONTRAST,CT ANGIOGRAPHY AORTO-ILEOFEMORAL RUNOFF WITH IV CONTRAST |
| CTA AORTO-ILEOFEMORAL RUNOFF W | CT ANGIOGRAPHY AORTO-ILEOFEMORAL RUNOFF WITH IV CONTRAST,CT ANGIOGRAPHY AORTO-ILEOFEMORAL RUNOFF WITH IV CONTRAST,CT Dual Energy |
| CTA AORTO-ILEOFEMORAL RUNOFF W | CT ANGIOGRAPHY AORTO-ILEOFEMORAL RUNOFF WITH IV CONTRAST,CT Dual Energy |
| CTA AORTO-ILEOFEMORAL RUNOFF W | CT ANGIOGRAPHY AORTO-ILEOFEMORAL RUNOFF WITH IV CONTRAST,CT Dual Energy,CT ANGIOGRAPHY AORTO-ILEOFEMORAL RUNOFF WITH IV CONTRAST |
| CTA AORTO-ILEOFEMORAL RUNOFF W | CT ANGIOGRAPHY AORTO-ILEOFEMORAL RUNOFF WITH IV CONTRAST,MM Reading |
| CTA AORTO-ILEOFEMORAL RUNOFF W | CT ANGIOGRAPHY AORTO-ILEOFEMORAL RUNOFF,CT ANGIOGRAPHY AORTO-ILEOFEMORAL RUNOFF |
| CTA AORTO-ILEOFEMORAL RUNOFF W | CT ANGIOGRAPHY AORTO-ILEOFEMORAL RUNOFF,CT ANGIOGRAPHY AORTO-ILEOFEMORAL RUNOFF,CT ANGIOGRAPHY AORTO-ILEOFEMORAL RUNOFF |
| CTA AORTO-ILEOFEMORAL RUNOFF WO | CT ANGIOGRAPHY AORTO-ILEOFEMORAL WO RUNOFF |
| CTA CAP | CT ANGIOGRAM CHEST ABD PELVIS |
| CTA CAP | CT ANGIOGRAM CHEST, ABDOMEN, AND PELVIS |
| CTA CAP | CT ANGIOGRAM CHEST, ABDOMEN, AND PELVIS WITH IV CONTRAST |
| CTA CAP | CT ANGIOGRAM CHEST, ABDOMEN, AND PELVIS WITH IV CONTRAST,CT ANGIOGRAM CHEST, ABDOMEN, AND PELVIS WITH IV CONTRAST |
| CTA CAP | CT ANGIOGRAM CHEST, ABDOMEN, AND PELVIS WITH IV CONTRAST,CT ANGIOGRAM CHEST, ABDOMEN, AND PELVIS WITH IV CONTRAST,CT ANGIOGRAM CHEST, ABDOMEN, AND PELVIS WITH IV CONTRAST |
| CTA CAP | CT ANGIOGRAM CHEST, ABDOMEN, AND PELVIS WITH IV CONTRAST,CT ANGIOGRAM CHEST, ABDOMEN, AND PELVIS WITH IV CONTRAST,CT Dual Energy |
| CTA CAP | CT ANGIOGRAM CHEST, ABDOMEN, AND PELVIS WITH IV CONTRAST,CT ANGIOGRAM CHEST, ABDOMEN, AND PELVIS WITH IV CONTRAST,MM Reading |
| CTA CAP | CT ANGIOGRAM CHEST, ABDOMEN, AND PELVIS WITH IV CONTRAST,CT Cardiac |
| CTA CAP | CT ANGIOGRAM CHEST, ABDOMEN, AND PELVIS WITH IV CONTRAST,CT Dual Energy |
| CTA CAP | CT ANGIOGRAM CHEST, ABDOMEN, AND PELVIS WITH IV CONTRAST,CT Dual Energy,CT ANGIOGRAM CHEST, ABDOMEN, AND PELVIS WITH IV CONTRAST |
| CTA CAP | CT ANGIOGRAM CHEST, ABDOMEN, AND PELVIS WITH IV CONTRAST,CT Vascular |
| CTA CAP | CT ANGIOGRAM CHEST, ABDOMEN, AND PELVIS WITH IV CONTRAST,MM Reading |
| CTA CAP | CT ANGIOGRAM CHEST, ABDOMEN, AND PELVIS,CT ANGIOGRAM CHEST, ABDOMEN, AND PELVIS |
| CTA CAP | CT ANGIOGRAM CHEST, ABDOMEN, AND PELVIS,CT ANGIOGRAM CHEST, ABDOMEN, AND PELVIS,CT ANGIOGRAM CHEST, ABDOMEN, AND PELVIS |
| CTA CAP | CT ANGIOGRAM CHEST, ABDOMEN, AND PELVIS,CT ANGIOGRAM CHEST, ABDOMEN, AND PELVIS,CT ANGIOGRAM CHEST, ABDOMEN, AND PELVIS,CT ANGIOGRAM CHEST, ABDOMEN, AND PELVIS |
| CTA CARDIAC STRUCT W QUANT EVALUATION | CTA CARDIAC STRUCT W QUANT EVALUATION |
| CTA CARDIAC STRUCT WO QUANT EVALUATION | CTA CARDIAC STRUCT WO QUANT EVALUATION |
| CTA CHEST PE W | CT ANGIOGRAM CHEST FOR PE WITH IV CONTRAST |
| CTA CHEST PE W | CT ANGIOGRAM CHEST FOR PE WITH IV CONTRAST,CT ANGIOGRAM CHEST FOR PE WITH IV CONTRAST |
| CTA CHEST PE W | CT ANGIOGRAM CHEST FOR PE WITH IV CONTRAST,CT ANGIOGRAM CHEST FOR PE WITH IV CONTRAST,CT ANGIOGRAM CHEST FOR PE WITH IV CONTRAST |
| CTA CHEST PE W | CT ANGIOGRAM CHEST FOR PE WITH IV CONTRAST,CT ANGIOGRAM CHEST FOR PE WITH IV CONTRAST,CT ANGIOGRAM CHEST FOR PE WITH IV CONTRAST,CT ANGIOGRAM CHEST FOR PE WITH IV CONTRAST |
| CTA CHEST PE W | CT ANGIOGRAM CHEST FOR PE WITH IV CONTRAST,CT ANGIOGRAM CHEST FOR PE WITH IV CONTRAST,CT ANGIOGRAM CHEST FOR PE WITH IV CONTRAST,CT ANGIOGRAM CHEST FOR PE WITH IV CONTRAST,CT ANGIOGRAM CHEST FOR PE WITH IV CONTRAST |
| CTA CHEST PE W | CT ANGIOGRAM CHEST FOR PE WITH IV CONTRAST,CT ANGIOGRAM CHEST FOR PE WITH IV CONTRAST,CT ANGIOGRAM CHEST FOR PE WITH IV CONTRAST,CT Dual Energy |
| CTA CHEST PE W | CT ANGIOGRAM CHEST FOR PE WITH IV CONTRAST,CT ANGIOGRAM CHEST FOR PE WITH IV CONTRAST,CT ANGIOGRAM CHEST FOR PE WITH IV CONTRAST,CT Dual Energy,CT ANGIOGRAM CHEST FOR PE WITH IV CONTRAST |
| CTA CHEST PE W | CT ANGIOGRAM CHEST FOR PE WITH IV CONTRAST,CT ANGIOGRAM CHEST FOR PE WITH IV CONTRAST,CT Dual Energy |
| CTA CHEST PE W | CT ANGIOGRAM CHEST FOR PE WITH IV CONTRAST,CT ANGIOGRAM CHEST FOR PE WITH IV CONTRAST,MM Reading |
| CTA CHEST PE W | CT ANGIOGRAM CHEST FOR PE WITH IV CONTRAST,CT Dual Energy |
| CTA CHEST PE W | CT ANGIOGRAM CHEST FOR PE WITH IV CONTRAST,CT Dual Energy,CT ANGIOGRAM CHEST FOR PE WITH IV CONTRAST |
| CTA CHEST PE W | CT ANGIOGRAM CHEST FOR PE WITH IV CONTRAST,MM Reading |
| CTA CHEST W | CT ANGIOGRAPHY C |
| CTA CHEST W | CT ANGIOGRAPHY CHEST |
| CTA CHEST W | CT ANGIOGRAPHY CHEST WITH IV CONTRAST |
| CTA CHEST W | CT ANGIOGRAPHY CHEST WITH IV CONTRAST,CT ANGIOGRAPHY CHEST WITH IV CONTRAST |
| CTA CHEST W | CT ANGIOGRAPHY CHEST WITH IV CONTRAST,CT ANGIOGRAPHY CHEST WITH IV CONTRAST,CT Dual Energy |
| CTA CHEST W | CT ANGIOGRAPHY CHEST WITH IV CONTRAST,CT Chest Pain |
| CTA CHEST W | CT ANGIOGRAPHY CHEST WITH IV CONTRAST,CT Dual Energy |
| CTA CHEST W | CT ANGIOGRAPHY CHEST WITH IV CONTRAST,MM Reading |
| CTA CHEST W | CT ANGIOGRAPHY CHEST,CT ANGIOGRAPHY CHEST |
| CTA CHEST W | CT ANGIOGRAPHY CHEST,CT ANGIOGRAPHY CHEST,CT ANGIOGRAPHY CHEST |
| CTA CHEST W | CT ANGIOGRAPHY CHEST,CT ANGIOGRAPHY CHEST,CT ANGIOGRAPHY CHEST,CT ANGIOGRAPHY CHEST |
| CTA CHEST W | CT ANGIOGRAPHY CHEST,CT ANGIOGRAPHY CHEST,CT CHEST WITH IV CONTRAST |
| CTA CHEST W | CT ANGIOGRAPHY CHEST,CT Vascular |
| CTA CORONARY ARTERY W QUANT EVALUATION | CTA CORONARY ARTERY W QUANT EVALUATION |
| CTA HEAD W | CT ANGIOGRAPHY H |
| CTA HEAD W | CT ANGIOGRAPHY H,CT ANGIOGRAPHY H |
| CTA HEAD W | CT ANGIOGRAPHY H,CT ANGIOGRAPHY H,CT ANGIOGRAPHY H |
| CTA HEAD W | CT ANGIOGRAPHY HEAD |
| CTA HEAD W | CT ANGIOGRAPHY HEAD WITH IV CONTRAST |
| CTA HEAD W | CT ANGIOGRAPHY HEAD WITH IV CONTRAST,CT ANGIOGRAPHY HEAD WITH IV CONTRAST |
| CTA HEAD W | CT ANGIOGRAPHY HEAD WITH IV CONTRAST,CT ANGIOGRAPHY HEAD WITH IV CONTRAST,CT ANGIOGRAPHY HEAD WITH IV CONTRAST |
| CTA HEAD W | CT ANGIOGRAPHY HEAD WITH IV CONTRAST,CT ANGIOGRAPHY HEAD WITH IV CONTRAST,CT ANGIOGRAPHY HEAD WITH IV CONTRAST,CT Dual Energy |
| CTA HEAD W | CT ANGIOGRAPHY HEAD WITH IV CONTRAST,CT ANGIOGRAPHY HEAD WITH IV CONTRAST,CT Dual Energy |
| CTA HEAD W | CT ANGIOGRAPHY HEAD WITH IV CONTRAST,CT ANGIOGRAPHY HEAD WITH IV CONTRAST,CT Dual Energy,CT ANGIOGRAPHY HEAD WITH IV CONTRAST |
| CTA HEAD W | CT ANGIOGRAPHY HEAD WITH IV CONTRAST,CT ANGIOGRAPHY HEAD WITH IV CONTRAST,MM Reading |
| CTA HEAD W | CT ANGIOGRAPHY HEAD WITH IV CONTRAST,CT Dual Energy |
| CTA HEAD W | CT ANGIOGRAPHY HEAD WITH IV CONTRAST,CT Dual Energy,CT ANGIOGRAPHY HEAD WITH IV CONTRAST |
| CTA HEAD W | CT ANGIOGRAPHY HEAD WITH IV CONTRAST,MM Reading |
| CTA HEAD W | CT ANGIOGRAPHY HEAD,CT ANGIOGRAPHY HEAD |
| CTA HEAD W | CT ANGIOGRAPHY HEAD,CT ANGIOGRAPHY HEAD,CT ANGIOGRAPHY HEAD |
| CTA HEAD W | CT ANGIOGRAPHY HEAD,CT ANGIOGRAPHY HEAD,CT ANGIOGRAPHY HEAD,CT ANGIOGRAPHY HEAD |
| CTA LOWER EX W | CT ANGIOGRAPHY L |
| CTA LOWER EX W | CT ANGIOGRAPHY LOWER EXTREMITIES |
| CTA LOWER EX W | CT ANGIOGRAPHY LOWER EXTREMITIES WITH IV CONTRAST |
| CTA LOWER EX W | CT ANGIOGRAPHY LOWER EXTREMITIES WITH IV CONTRAST,CT Dual Energy |
| CTA NECK W | CT ANGIOGRAPHY N |
| CTA NECK W | CT ANGIOGRAPHY NECK |
| CTA NECK W | CT ANGIOGRAPHY NECK WITH IV CONTRAST |
| CTA NECK W | CT ANGIOGRAPHY NECK WITH IV CONTRAST,CT ANGIOGRAPHY NECK WITH IV CONTRAST |
| CTA NECK W | CT ANGIOGRAPHY NECK WITH IV CONTRAST,CT ANGIOGRAPHY NECK WITH IV CONTRAST,CT ANGIOGRAPHY NECK WITH IV CONTRAST,CT Dual Energy |
| CTA NECK W | CT ANGIOGRAPHY NECK WITH IV CONTRAST,CT ANGIOGRAPHY NECK WITH IV CONTRAST,CT Dual Energy |
| CTA NECK W | CT ANGIOGRAPHY NECK WITH IV CONTRAST,CT Dual Energy |
| CTA NECK W | CT ANGIOGRAPHY NECK WITH IV CONTRAST,MM Reading |
| CTA NECK W | CT ANGIOGRAPHY NECK,CT ANGIOGRAPHY NECK |
| CTA NECK W | CT ANGIOGRAPHY NECK,CT ANGIOGRAPHY NECK,CT ANGIOGRAPHY NECK |
| CTA PELV W | CT ANGIOGRAPHY PELVIS |
| CTA PELV W | CT ANGIOGRAPHY PELVIS WITH IV CONTRAST |
| CTA PELV W | CT ANGIOGRAPHY PELVIS,CT ANGIOGRAPHY PELVIS |
| CTA UPPER EX W | CT ANGIOGRAPHY UPPER EXTREMITY |
| CTA UPPER EX W | CT ANGIOGRAPHY UPPER EXTREMITY WITH IV CONTRAST |
| CTA UPPER EX W | CT ANGIOGRAPHY UPPER EXTREMITY WITH IV CONTRAST,CT ANGIOGRAPHY UPPER EXTREMITY WITH IV CONTRAST |
| CTA UPPER EX W | CT ANGIOGRAPHY UPPER EXTREMITY WITH IV CONTRAST,CT Dual Energy |
| CT-INTRA OP | CT-INTRA OP |
| FLUORO FLUOROSCOPY LESS THAN 1 HOUR | FLUORO FLUOROSCOPY LESS THAN 1 HOUR |
| HEPATIC LUNG PERFUSION STUDY W-SHUNT CALCULATIONS | HEPATIC LUNG PERFUSION STUDY W-SHUNT CALCULATIONS |
| IR ADRENAL VENOUS SAMPLING | IR ADRENAL VENOUS SAMPLING |
| IR ARTERIOGRAM ABDOM AND PELVIC WITH R/O'S | IR ARTERIOGRAM ABDOM AND PELVIC WITH R/O'S |
| IR ARTERIOGRAM ABDOM W/ NON-SELECTIVE PELVIS | IR ARTERIOGRAM ABDOM W/ NON-SELECTIVE PELVIS |
| IR ARTERIOGRAM VISCERAL | IR ARTERIOGRAM VISCERAL |
| IR BALLOON-OCCLUDED RETROGRADE TRANSVENOUS OBLITERATION (BRTO) | IR BALLOON-OCCLUDED RETROGRADE TRANSVENOUS OBLITERATION (BRTO) |
| IR BILLIARY DRAIN CHECK | IR BILLIARY DRAIN CHECK |
| IR CHOLECYSTOSTOMY TUBE PLACEMENT | IR CHOLECYSTOSTOMY TUBE PLACEMENT |
| IR CT ABLATION | IR CT ABLATION |
| IR CT ABLATION | IR CT Ablation Cryo |
| IR CT ABLATION | IR CT Ablation RFA |
| IR CT ABLATION | IR CT CRYO ABLATION |
| IR CT ABLATION | IR CT MICROWAVE ABLATION |
| IR CT ABLATION | IR CT MICROWAVE ABLATION,IR CT MICROWAVE ABLATION |
| IR CT ABLATION | IR CT RFA ABLATION |
| IR CT ABSCESSOGRAM TUBE CHECK | IR ABSCESSOGRAM |
| IR CT ABSCESSOGRAM TUBE CHECK | IR ABSCESSOGRAM TUBE CHECK |
| IR CT ABSCESSOGRAM TUBE CHECK | IR CT ABSCESSOGR |
| IR CT ABSCESSOGRAM TUBE CHECK | IR CT ABSCESSOGRAM TUBE CHECK |
| IR CT ABSCESSOGRAM TUBE CHECK | IR CT ABSCESSOGRAM TUBE CHECK,IR CT ABSCESSOGRAM TUBE CHECK |
| IR CT BIOPSY | IR CT BIOPSY |
| IR CT BIOPSY | IR CT BIOPSY,IR CT BIOPSY |
| IR CT BIOPSY | IR CT BIOPSY,IR CT BIOPSY,IR CT BIOPSY |
| IR CT CERVICAL SPINE WITHOUT CONTRAST | IR CT CERVICAL SPINE WITHOUT CONTRAST |
| IR CT DRAINAGE | IR CT DRAINAGE |
| IR CT DRAINAGE | IR CT DRAINAGE,IR CT DRAINAGE |
| IR CT DRAINAGE | IR CT DRAINAGE,IR CT GUIDED ASPIRATION |
| IR CT GUIDED ASPIRATION | IR CT GUIDED ASP |
| IR CT GUIDED ASPIRATION | IR CT GUIDED ASPIRATION |
| IR CT GUIDED ASPIRATION | IR CT GUIDED ASPIRATION,IR CT GUIDED ASPIRATION |
| IR CT GUIDED ASPIRATION | IR CT GUIDED ASPIRATION,IR CT GUIDED ASPIRATION,IR CT GUIDED ASPIRATION,IR CT GUIDED ASPIRATION |
| IR CT GUIDED FIDUCIAL PLACEMENT | IR CT GUIDED FIDUCIAL PLACEMENT |
| IR CT IRREVERSIBLE ELECTROPORATION ABLATION | IR CT IRREVERSIBLE ELECTROPORATION ABLATION |
| IR CT IRREVERSIBLE ELECTROPORATION ABLATION | IR CT Irreversible Electroporation Ablation |
| IR CT L-SPINE WO | IR CT LUMBAR SPINE WITHOUT CONTRAST |
| IR CT NANOKNIFE | IR CT NANOKNIFE |
| IR CT NANOKNIFE | IR CT NANOKNIFE ABLATION |
| IR CT PULSED ELECTRICAL FIELD ABLATION | IR CT PULSED ELECTRICAL FIELD ABLATION |
| IR CT THORACIC WO | IR CT THORACIC WITHOUT CONTRAST |
| IR EMBOLIZATION | IR EMBOLIZATION |
| IR FLUORO NEEDLE PLACEMENT | IR FLUORO NEEDLE PLACEMENT |
| IR G TUBE EXCHANGE | IR G TUBE EXCHANGE |
| IR G TUBE PLACEMENT | IR G TUBE PLACEMENT |
| IR G-J TUBE PLACEMENT | IR G-J TUBE PLACEMENT |
| IR J TUBE PLACEMENT | IR J TUBE PLACEMENT |
| IR KYPHOPLASTY | IR KYPHOPLASTY |
| IR LIVER BIOPSY RANDOM | IR LIVER BIOPSY RANDOM |
| IR MINOR PROCEDURE | IR MINOR PROCEDURE |
| IR MINOR PROCEDURE | IR MINOR PROCEDURE,IR MINOR PROCEDURE |
| IR MYELOGRAM COMBINED SPINE | IR MYELOGRAM COMBINED SPINE |
| IR MYELOGRAM LUMBAR SPINE | IR MYELOGRAM LUMBAR SPINE |
| IR NEPHROSTOMY PRIMARY BILATERAL | IR NEPHROSTOMY PRIMARY BILATERAL |
| IR NEPHROURETERAL STENT EXCHANGE BILATERAL | IR NEPHROURETERAL STENT EXCHANGE BILATERAL |
| IR NEPHROURETERAL STENT EXCHANGE UNILATERAL | IR NEPHROURETERAL STENT EXCHANGE UNILATERAL |
| IR NEURO FLUORO | IR NEURO FLUORO |
| IR NON-TUNNELED CENTRAL VENOUS CATHETER | IR NON-TUNNELED CENTRAL VENOUS CATHETER |
| IR PARACENTESIS WITH NON-TUNNELED DRAIN PLACEMENT | IR PARACENTESIS WITH NON-TUNNELED DRAIN PLACEMENT |
| IR PICC CHANGE | IR PICC CHANGE |
| IR PICC INSERTION | IR PICC INSERTION |
| IR PLEUREX CATHETER PLACEMENT PLEURAL LEFT | IR PLEUREX CATHETER PLACEMENT PLEURAL LEFT |
| IR PORTAL VEIN EMBOLIZATION | IR PORTAL VEIN EMBOLIZATION |
| IR RADIOEMBOLIZATION W/ THERASPHERE | IR RADIOEMBOLIZATION W/ THERASPHERE |
| IR RADIOEMBOLIZATION WORKUP/ MAA HEPATIC LUNG (MAPPING) | IR RADIOEMBOLIZATION WORKUP/ MAA HEPATIC LUNG (MAPPING) |
| IR RENAL BIOPSY PERCUTANEOUS | IR RENAL BIOPSY PERCUTANEOUS |
| IR REPAIR INFRARENAL AAA OR DISSECTION | IR REPAIR INFRARENAL AAA OR DISSECTION |
| IR SINUS FISTULA TRACT INJECTION | IR SINUS FISTULA TRACT INJECTION |
| IR SPINAL PUNCTURE | IR SPINAL PUNCTURE |
| IR SUPRAPUBIC CATHETER | IR SUPRAPUBIC CATHETER |
| IR SUPRAPUBIC CATHETER PLACEMENT | IR SUPRAPUBIC CATHETER PLACEMENT |
| IR THORACENTESIS | IR THORACENTESIS |
| IR TIPS PRIMARY | IR TIPS PRIMARY |
| IR TIPS REVISION | IR TIPS REVISION |
| IR ULTRASOUND BIOPSY | IR ULTRASOUND BIOPSY |
| IR US GUIDED ASPIRATION | IR US GUIDED ASPIRATION |
| IR US PARACENTESIS W/O DRAIN | IR US PARACENTESIS W/O DRAIN |
| IR US PARACENTESIS W-O DRAIN | IR US PARACENTESIS W-O DRAIN |
| IR VENOGRAM EXTREMITY UNILATERAL | IR VENOGRAM EXTREMITY UNILATERAL |
| IR VENOGRAM INFERIOR VENA CAVA | IR VENOGRAM INFERIOR VENA CAVA |
| Localizer | CT SCAN FOR LOCA |
| Localizer | CT SCAN FOR LOCALIZATION |
| Localizer | CT SCAN FOR LOCALIZATION WITHOUT IV CONTRAST |
| Localizer | CT SCAN FOR LOCALIZATION WITHOUT IV CONTRAST,CT Dual Energy |
| Localizer | CT SCAN FOR LOCALIZATION WITHOUT IV CONTRAST,MM Reading |
| Localizer | CT SCAN FOR LOCALIZATION,CT SCAN FOR LOCALIZATION |
| Localizer | CT SCAN FOR LOCALIZATION,CT SCAN FOR LOCALIZATION,CT SCAN FOR LOCALIZATION |
| MM Reading | MM Reading |
| MRI ABDOMEN WO AND W IV CONT | MRI ABDOMEN WO AND W IV CONT |
| MRI BRAIN WITHOUT AND WITH IV CONTRAST | MRI BRAIN WITHOUT AND WITH IV CONTRAST |
| MRI BRAIN WO AND W IV CONT | MRI BRAIN WO AND W IV CONT |
| NM BONE MARROW SCAN | NM BONE MARROW SCAN |
| NM BONE SPECT | NM BONE SPECT |
| NM BREMSSTRAHLUNG POST RADIOEMBOLIZATION IMAGING OF ABD/CHES | NM BREMSSTRAHLUNG POST RADIOEMBOLIZATION IMAGING OF ABD/CHES |
| NM BRMSSTRLNG POST RADIOEMBO IMG ABD/CHEST WO OR W SPECT CT | NM BRMSSTRLNG POST RADIOEMBO IMG ABD/CHEST WO OR W SPECT CT |
| NM CARDIAC AMYLOID SCAN WO OR W SPECT CT | NM CARDIAC AMYLOID SCAN WO OR W SPECT CT |
| NM CARDIAC REST AND STRESS COMBINED | NM CARDIAC REST AND STRESS COMBINED |
| NM CERETEC WHITE BLOOD CELL | NM CERETEC WHITE BLOOD CELL |
| NM CERETEC WHITE BLOOD CELL SINGLE DAY IMAGING | NM CERETEC WHITE BLOOD CELL SINGLE DAY IMAGING |
| NM CERETEC WHITE BLOOD CELL SINGLE DAY IMG WO OR W SPECT CT | NM CERETEC WHITE BLOOD CELL SINGLE DAY IMG WO OR W SPECT CT |
| NM EXERCISE CARDIAC STRESS WITH NUCLEAR IMAGING & CT NUCLEAR | NM EXERCISE CARDIAC STRESS WITH NUCLEAR IMAGING & CT NUCLEAR |
| NM GALLIUM SCAN | NM GALLIUM SCAN |
| NM GALLIUM SCAN SINGLE DAY IMAGING | NM GALLIUM SCAN SINGLE DAY IMAGING |
| NM HEPATIC LUNG PERFUSION STUDY WO OR W SPECT CT | NM HEPATIC LUNG PERFUSION STUDY WO OR W SPECT CT |
| NM HEPATOBILIARY SCAN | NM HEPATOBILIARY SCAN |
| NM HEPATOBILIARY SCAN WITH CCK WO OR W SPECT CT | NM HEPATOBILIARY SCAN WITH CCK WO OR W SPECT CT |
| NM I-123 MIBG TUMOR SCAN | NM I-123 MIBG TUMOR SCAN |
| NM I-123 MIBG TUMOR SCAN 2 OR MORE DAYS IMAGING | NM I-123 MIBG TUMOR SCAN 2 OR MORE DAYS IMAGING |
| NM I-123 MIBG TUMOR SCN 2 OR MORE DAYS IMG WO OR W SPECT CT | NM I-123 MIBG TUMOR SCN 2 OR MORE DAYS IMG WO OR W SPECT CT |
| NM I-123 SURVEILLANCE TOTAL BODY SCAN | NM I-123 SURVEILLANCE TOTAL BODY SCAN |
| NM I123 THYROID UPTAKE SCAN | NM I123 THYROID UPTAKE SCAN |
| NM I-123 WHOLE BODY SCAN | NM I-123 WHOLE BODY SCAN |
| NM I-123 WHOLE BODY SCAN WO OR W SPECT CT | NM I-123 WHOLE BODY SCAN WO OR W SPECT CT |
| NM I-131 7 DAY POST THERAPY SCAN | NM I-131 7 DAY POST THERAPY SCAN |
| NM I-131 Surveillance Total Body Scan | NM I-131 Surveillance Total Body Scan |
| NM I131 TOTAL BODY SCAN | NM I131 TOTAL BODY SCAN |
| NM IN111 WHITE BLOOD CELL SCAN | NM IN111 WHITE BLOOD CELL SCAN |
| NM IN111 WHITE BLOOD CELL SCAN 2 OR MORE DAY IMAGING | NM IN111 WHITE BLOOD CELL SCAN 2 OR MORE DAY IMAGING |
| NM LIVER SPLEEN SCAN | NM LIVER SPLEEN SCAN |
| NM LYMPHANGIOGRAM | NM LYMPHANGIOGRAM |
| NM LYMPHANGIOGRAM NON-BREAST WO OR W SPECT CT | NM LYMPHANGIOGRAM NON-BREAST WO OR W SPECT CT |
| NM OCTREOSCAN | NM OCTREOSCAN |
| NM OCTREOSCAN 2 OR MORE DAY IMAGING | NM OCTREOSCAN 2 OR MORE DAY IMAGING |
| NM PARATHYROID SCAN AND SPECT-CT | NM PARATHYROID SCAN AND SPECT-CT |
| NM PARATHYROID SCAN SPECT | NM PARATHYROID SCAN SPECT |
| NM PHARMACOLOGIC CARDIAC STRESS WITH NUCLEAR IMAGING | NM PHARMACOLOGIC CARDIAC STRESS WITH NUCLEAR IMAGING |
| NM Pharmacologic Cardiac Stress with Nuclear Imaging | NM Pharmacologic Cardiac Stress with Nuclear Imaging |
| NM POST THERAPY SCAN WITHOUT OR WITH SPECT-CT | NM POST THERAPY SCAN WITHOUT OR WITH SPECT-CT |
| NM RESTING CARDIAC SCAN - TECHNETIUM | NM RESTING CARDIAC SCAN - TECHNETIUM |
| NM RESTING CARDIAC SCAN - THALLIUM | NM RESTING CARDIAC SCAN - THALLIUM |
| NM REST-STRESS CARDIAC W VIABILITY | NM REST-STRESS CARDIAC W VIABILITY |
| NM STRESS CARDIAC SCAN - TECHNETIUM | NM STRESS CARDIAC SCAN - TECHNETIUM |
| NM TOTAL BODY BONE SCAN | NM TOTAL BODY BONE SCAN |
| NM TOTAL BODY BONE SCAN WO OR W SPECT-CT | NM TOTAL BODY BONE SCAN WO OR W SPECT-CT |
| NM TRIPLE PHASE BONE SCAN | NM TRIPLE PHASE BONE SCAN |
| NR CT BIOPSY | NR CT BIOPSY |
| NR CT BIOPSY NEURO | NR CT BIOPSY NEURO |
| NR CT DRAINAGE | NR CT DRAINAGE |
| NR CT MAXIOFACIAL POST CISTERNOGRAPHY | NR CT MAXIOFACIAL POST CISTERNOGRAPHY |
| NR CT MAXIOFACIAL PRE CISTERNOGRAPHY | NR CT MAXIOFACIAL PRE CISTERNOGRAPHY |
| NR CT MYELOGRAM | NR CT MYELOGRAM |
| NR CT MYELOGRAM | NR CT MYELOGRAM,NR CT MYELOGRAM |
| NR DISK BIOPSY FLUORO | NR DISK BIOPSY FLUORO |
| NR Disk Biopsy Fluoro | NR Disk Biopsy Fluoro |
| NR EPIDURAL | NR EPIDURAL |
| NR MYELOGRAM TOTAL SPINE | NR MYELOGRAM TOTAL SPINE |
| NR NERVE BLOCK | NR NERVE BLOCK |
| PET CT Cardiac Resting - Rubidium | PET CT Cardiac Resting - Rubidium |
| PET-CT BREAST MONITOR EVAL TO TREATMENT | PET-CT BREAST MONITOR EVAL TO TREATMENT |
| PET-CT CARDIAC SARCOID PROTOCOL | PET-CT CARDIAC SARCOID PROTOCOL |
| PET-CT EVALUATION OF TREATMENT BRAIN | PET-CT EVALUATION OF TREATMENT BRAIN |
| PET-CT METABOLIC BRAIN SEIZURE | PET-CT METABOLIC BRAIN SEIZURE |
| PET-CT MYOCARDIAC VIABILITY | PET-CT MYOCARDIAC VIABILITY |
| PET-CT MYOCARDIAL VIABILITY | PET-CT MYOCARDIAL VIABILITY |
| PET-CT RESEARCH BRAIN | PET-CT RESEARCH BRAIN |
| PET-CT SKULL BASE-MID THIGH INITIAL STAGING LYMPHOMA | PET-CT SKULL BASE-MID THIGH INITIAL STAGING LYMPHOMA |
| PET-CT SKULL BASE-MID THIGH RESTAGING NEUROENDOCRINE TUMOR | PET-CT SKULL BASE-MID THIGH RESTAGING NEUROENDOCRINE TUMOR |
| PET-CT SKULL BASE-MID THIGH RESTAGING SMALL CELL LUNG | PET-CT SKULL BASE-MID THIGH RESTAGING SMALL CELL LUNG |
| PET-CT SKULL BASE-MID TUMOR SURVEY | PET-CT SKULL BASE-MID TUMOR SURVEY |
| PET-CT STAGING RESTAGING BREAST | PET-CT STAGING RESTAGING BREAST |
| PET-CT TOTAL BODY TUMOR SURVEY | PET-CT TOTAL BODY TUMOR SURVEY |
| PET-CT W CONCURRENT DIAGNOSTIC CT-CHEST/ABD/PELVIS W CON | PET-CT W CONCURRENT DIAGNOSTIC CT-CHEST/ABD/PELVIS W CON |
| PET-CT WHOLEBODY INITAL STAGING CERVICAL | PET-CT WHOLEBODY INITAL STAGING CERVICAL |
| PET-CT WHOLEBODY INITAL STAGING THYROID | PET-CT WHOLEBODY INITAL STAGING THYROID |
| PET-CT WHOLEBODY INITIAL STAGING COLORECTAL CA | PET-CT WHOLEBODY INITIAL STAGING COLORECTAL CA |
| PET-CT WHOLEBODY INITIAL STAGING ESOPHAGEAL CA | PET-CT WHOLEBODY INITIAL STAGING ESOPHAGEAL CA |
| PET-CT WHOLEBODY INITIAL STAGING HEAD NECK CA | PET-CT WHOLEBODY INITIAL STAGING HEAD NECK CA |
| PET-CT WHOLEBODY INITIAL STAGING LYMPHOMA | PET-CT WHOLEBODY INITIAL STAGING LYMPHOMA |
| PET-CT WHOLEBODY INITIAL STAGING MELANOMA | PET-CT WHOLEBODY INITIAL STAGING MELANOMA |
| PET-CT WHOLEBODY INITIAL STAGING MYELOMA | PET-CT WHOLEBODY INITIAL STAGING MYELOMA |
| PET-CT WHOLEBODY INITIAL STAGING NON SMALL CELL LUNG | PET-CT WHOLEBODY INITIAL STAGING NON SMALL CELL LUNG |
| PET-CT Wholebody Initial Staging Ovarian | PET-CT Wholebody Initial Staging Ovarian |
| PET-CT WHOLEBODY INITIAL STAGING PANCREAS | PET-CT WHOLEBODY INITIAL STAGING PANCREAS |
| PET-CT WHOLEBODY INITIAL STAGING SMALL CELL LUNG | PET-CT WHOLEBODY INITIAL STAGING SMALL CELL LUNG |
| PET-CT WHOLEBODY INITIAL STAGING SOFT TISSUE SARCOMA | PET-CT WHOLEBODY INITIAL STAGING SOFT TISSUE SARCOMA |
| PET-CT WHOLEBODY RESTAGING CERVICAL | PET-CT WHOLEBODY RESTAGING CERVICAL |
| PET-CT WHOLEBODY RESTAGING COLORECTAL CA | PET-CT WHOLEBODY RESTAGING COLORECTAL CA |
| PET-CT WHOLEBODY RESTAGING ESOPHAGEAL CA | PET-CT WHOLEBODY RESTAGING ESOPHAGEAL CA |
| PET-CT WHOLEBODY RESTAGING FOLLICULAR THYROID CA | PET-CT WHOLEBODY RESTAGING FOLLICULAR THYROID CA |
| PET-CT WHOLEBODY RESTAGING HEAD NECK CA | PET-CT WHOLEBODY RESTAGING HEAD NECK CA |
| PET-CT WHOLEBODY RESTAGING LYMPHOMA | PET-CT WHOLEBODY RESTAGING LYMPHOMA |
| PET-CT WHOLEBODY RESTAGING MELANOMA | PET-CT WHOLEBODY RESTAGING MELANOMA |
| PET-CT WHOLEBODY RESTAGING MYELOMA | PET-CT WHOLEBODY RESTAGING MYELOMA |
| PET-CT WHOLEBODY RESTAGING NON SMALL CELL LUNG | PET-CT WHOLEBODY RESTAGING NON SMALL CELL LUNG |
| PET-CT WHOLEBODY RESTAGING OVARIAN | PET-CT WHOLEBODY RESTAGING OVARIAN |
| REQUEST FOR INTERVENTIONAL RADIOLOGY | REQUEST FOR INTERVENTIONAL RADIOLOGY |
| SIRSPHERES THERAPEUTIC IMAGING | SIRSPHERES THERAPEUTIC IMAGING |
| X-RAY CHEST 1 VIEW AP (PORTABLE) | X-RAY CHEST 1 VIEW AP (PORTABLE) |
| X-RAY HIP BILATERAL AND AP VIEW OF PELVIS | X-RAY HIP BILATERAL AND AP VIEW OF PELVIS |
